# The Effects of Latitudinal Gradients, Climatic Anomalies, and Size‐Selective Harvesting on the Adaptive Potential of an Intertidal Gastropod

**DOI:** 10.1111/eva.70159

**Published:** 2025-09-25

**Authors:** Erica S. Nielsen, Samuel Walkes, Jacqueline L. Sones, Phillip B. Fenberg, David A. Paz‐García, Richard K. Grosberg, Eric Sanford, Rachael A. Bay

**Affiliations:** ^1^ Department of Evolution and Ecology University of California Davis Davis California USA; ^2^ The Nature Conservancy Sacramento California USA; ^3^ Bodega Marine Laboratory University of California Davis Bodega Bay California USA; ^4^ Bodega Marine Reserve University of California Davis Bodega Bay California USA; ^5^ School of Ocean and Earth Sciences, National Oceanography Centre Southampton University of Southampton Southampton UK; ^6^ Laboratorio de Genética Para la Conservación Centro de Investigaciones Biológicas del Noroeste (CIBNOR) La Paz Baja California Sur Mexico

**Keywords:** conservation genomics, eco‐evolutionary dynamics, genotype‐association, *Lottia gigantea*, population adaptive potential, seascape genomics, species range shift

## Abstract

Coastal organisms live in a dynamic environment where a myriad of environmental stressors, including climate change, ocean acidification, and human harvesting, act on variable spatio‐temporal scales. Each of these stressors may impose unique selective forces on a population, shaping a species' adaptive potential and its ability to persist under future climatic conditions. Genomic investigations of adaptive responses to environmental and anthropogenic disturbances remain rare, especially in marine systems. Here, we use whole genome sequencing data from the owl limpet, *Lottia gigantea* , and outlier detection methods to pinpoint signals of selection (1) across long‐standing environmental gradients spanning the species' distribution, (2) at the poleward edge of the species' range where it experienced a recent expansion, and (3) between sites vulnerable to or protected from human size‐selective harvesting within California. Loci associated with environmental gradients across the entire range show the strongest differentiation at the southern end of the species' range, potentially driven by adaptation to sea surface temperature and pH. Additional *ad‐hoc* outlier analyses revealed a distinct set of loci potentially under selection in the expanded range, with different functional roles than the range‐wide outliers. Despite demographic models suggesting that protection from harvesting has a positive impact on the abundance of large individuals, we did not find strong signals of selection or changes in genetic diversity between sites differing in harvesting vulnerability. Our findings suggest that range‐wide environmental selective signals established over longer time scales are distinct from those imposed by climatic anomalies at finer spatio‐temporal scales. We found that climatic variation has a stronger selective imprint than human harvesting, and thus conservation interventions should consider prioritizing the maintenance of climate‐related adaptive potential. Understanding how climatic trends and anomalies interact with anthropogenic pressures will allow us to make more informed decisions to sustain the evolutionary capacity of *L. gigantea* and other key coastal species.

## 1 Introduction

Characterizing intraspecific adaptive potential of natural populations is increasingly important as threats to biodiversity such as climate change, harvesting pressure, and habitat degradation continue to harm species and ecosystems (Gissi et al. 2021; Munday et al. 2013). Some of the most pressing questions within evolutionary ecology and conservation are whether adaptive evolution can keep pace with the rate of selection, and if evolutionary adaptations from long‐standing environmental selection are also beneficial for coping with rapid climate change (Hill et al. 2011). Understanding how different populations are locally adapted to environmental conditions is essential to predict how they will respond to future changes such as increasing ocean temperature and acidification (Donelson et al. 2019; Kelly et al. 2012; Vargas et al. 2017). Assessments of intraspecific adaptive capacity are critical to inform conservation interventions designed to help natural populations cope with future change (Flanagan et al. 2018; Funk et al. 2019; Nicotra et al. 2015). It is also important to consider that species must respond to multiple and interacting environmental and anthropogenic factors, which often occur with different temporal cadences. However, most evaluations of population adaptive potential focus on range‐wide selection to long‐term climatic trends, without accounting for potentially interacting impacts such as human harvesting or climatic anomalies (Vázquez et al. 2017).

Climatic anomalies (i.e., short‐term deviations from a long‐term climatic baseline) such as marine heatwaves can impose demographic changes such as large‐scale die‐offs or range expansions, which can have distinct selection pressures and evolutionary consequences (Donelson et al. 2019; Harvey et al. 2022). Rapid evolution can occur in response to novel environments during a range expansion (Colautti and Lau 2015; Lucek et al. 2014; Lustenhouwer et al. 2018), with the knock‐on effect of leading‐edge populations having higher fitness, further facilitating future poleward expansions (Miller et al. 2020). Marine heatwaves can alter allele frequencies alongside long‐term climatic trends. For example, there was a rapid poleward shift of warm‐adapted alleles in the kelp, *Ecklonia radiata*, across 200 km of coastline in Australia following a marine heatwave (Coleman et al. 2020). Thus, the adaptive potential of range‐edge populations will not only be influenced by long‐term climatic trends, but also by climatic anomalies and associated demographic changes (Melero et al. 2022; Pershing et al. 2018). For instance, the poleward‐shifting damselfly, *Coenagrion scitulum*, exhibits increased genetic differentiation from the species' core due to genetic drift after colonization (Swaegers et al. 2014). However, genetic variation often reflects both past and contemporary environments, as there is a time lag between demographic changes and the genomic change they cause (Epps and Keyghobadi 2015). Disentangling how environmental variation over different spatio‐temporal scales shapes intraspecific genomic patterns is difficult, as genetic composition depends on multiple interacting factors such as the strength of selection, mutation rate, effective population size, and gene flow (Epps and Keyghobadi 2015; Gargiulo et al. 2024).

Anthropogenic pressures may also impose selection on populations, further influencing how they respond to climate anomalies and trends. For example, harvesting may alter phenotypic traits such as body mass and age at maturation (Hamilton et al. 2007; Uusi‐Heikkilä et al. 2015) and potentially have synergistic effects on population dynamics when combined with climate change (Harley and Rogers‐Bennett 2004). Harvesting can also have genomic effects, with several studies showing that harvested populations have lower genetic diversity if population sizes decrease due to overharvesting (Allendorf et al. 2008; Pinsky and Palumbi 2014; Sadler et al. 2023). While several empirical studies show that harvesting decreases abundance and biomass of exploited marine species (Baliwe et al. 2022; Harmelin‐Vivien et al. 2008; Marra et al. 2017), genetic diversity does not always decrease in harvested sites (Benestan et al. 2023; Figuerola‐Ferrando et al. 2023; Yorisue et al. 2020). The duration of the time‐lag between harvesting causing demographic changes (i.e., population declines) and genomic changes (i.e., allele frequency shifts) could explain some of these mixed results (Benestan et al. 2023; Laugen et al. 2014; Lourenço et al. 2017). The strength of environmental selection may also outweigh that of harvesting, weakening the relationship between harvesting and genomic variation (Cameron et al. 2013). Studies assessing species responses to both harvesting and environmental pressures can reveal how selection across different spatio‐temporal scales affects genetic diversity and adaptive potential.

Here we investigate genomic signals of local selection on the owl limpet, *Lottia gigantea* . *Lottia gigantea* is ecologically important as it alters available space within the rocky intertidal zone through territorial grazing (Lindberg et al. 1998; Stimson 1970). *Lottia gigantea* is protandric hermaphrodite, generally changing sex from male to female as they age (Kido and Murray 2003), and size‐selective harvesting of *L. gigantea* can lead to smaller individuals that generally grow more slowly and change sex at a smaller size (Fenberg and Roy 2012; Pombo and Escofet 1996; Roy et al. 2003; Sagarin et al. 2007). Harvested sites were also found to have higher abundances of *L. gigantea* than non‐harvested sites, likely because they were composed of smaller individuals which take up less space (Fenberg and Rivadeneira 2011). No significant difference in the genetic diversity of *L. gigantea* between exploited and protected sites was found using six microsatellite markers (Fenberg et al. 2010).

**FIGURE 1:**
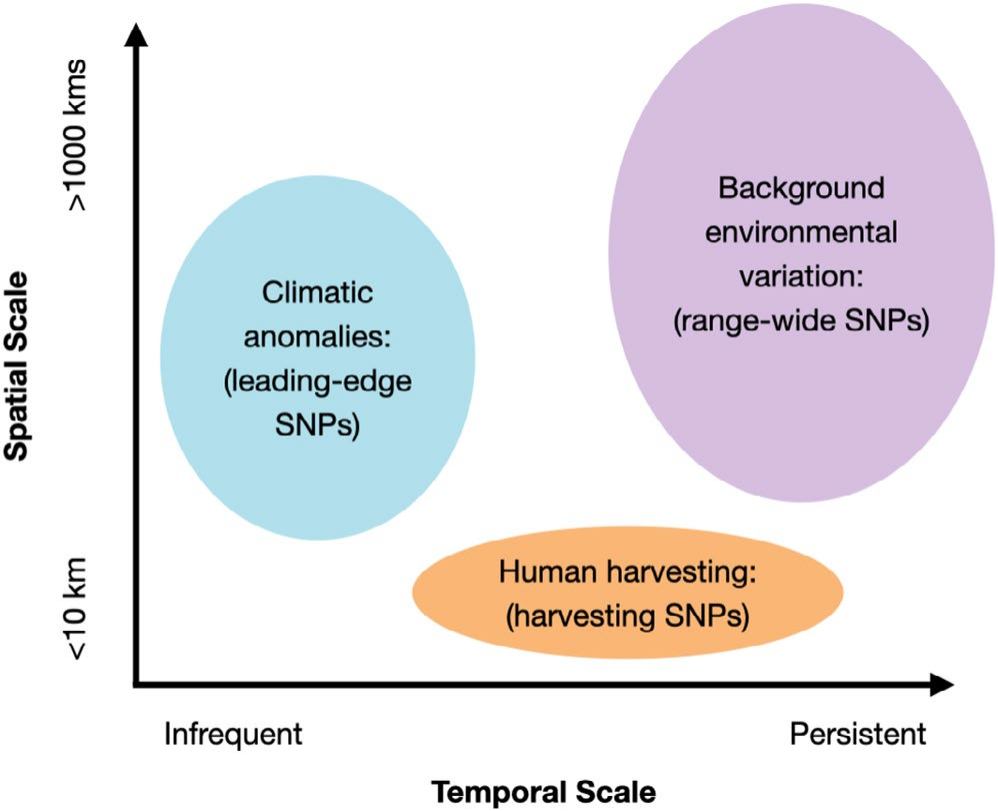
Diagram of the methods used to test how genomic selection and demographic patterns are shaped by the following environmental/anthropogenic forces: An environmental gradient, climatic anomalies, and size‐selective harvesting. These three forces occur at different spatio‐temporal resolutions, with environmental clines existing over the largest spatial and temporal scales. Climatic anomalies such as heatwaves are more temporally punctuated compared to most harvesting efforts, but often have a broader spatial impact than the site‐specific harvesting that occurs for many non‐commercially exploited coastal organisms. We ran outlier detection analyses to identify adaptive SNPs associated with each type of selective force, with the nomenclature shown in parentheses.

*Lottia gigantea* underwent a range expansion associated with marine heatwaves and an El Niño event during 2014–2016 within the northeast Pacific (Sanford et al. 2019). Anomalous water temperatures and ocean currents increased recruitment of *L. gigantea* within the poleward expanded range, with ongoing reproduction and recruitment since (Sanford et al., in prep.). While the historical range of *L. gigantea* extended even farther north (based on museum specimens from ~41° N collected in 1889, 1935, 1957, and 1963), this is the first time a stable population with reliable annual recruitment has occurred north of ~38° N in over 20 years of monitoring (Fenberg and Rivadeneira 2011; Sanford et al. 2019). This work is an extension of a genomic study that identified the neutral population structure of *L. gigantea* (Nielsen et al. 2024), which revealed that the range expansion is most consistent with a ‘pushed wave’ (Miller et al. 2020)– where recruits to the expansion edge come from a large gene pool across the species' range (see Nielsen et al. 2024 for details). The present study expands on those initial analyses to examine the adaptive patterns associated with this pushed‐wave expansion event. We test how ecological drivers at different scales shape genomic patterns using outlier identification tests, creating three independent panels of single nucleotide polymorphisms (SNPs) that capture adaptive responses to: (1) long‐standing environmental variation across the geographic range, (2) pulses of extreme climatic events leading to a northern range expansion, and (3) human harvesting. Long‐term averages in environmental variation extend over larger spatio‐temporal scales, representing historical/prolonged selection across the geographic range, whereas climatic anomalies have shorter and more variable spatio‐temporal extents, representing ‘pulse’ selection events (Figure 1; Harris et al. 2018). Size‐selective harvesting may be temporally more consistent than climatic anomalies, but harvesting pressure can vary over smaller spatial scales due to the presence of protected areas and local inaccessibility (Figure 1). We utilize this ideal system to investigate how harvesting pressure interacts with climate anomalies and environmental gradients to drive selective landscapes in a range‐shifting species, using genomics to offer comprehensive conservation and adaptive species management inferences.

Specifically, we address the following questions: (1) What are the genomic selective signals of the recent poleward recruitment event and how do they compare with background selection across the entire range?; (2) What environmental variables are associated with genomic variation?; (3) Does vulnerability to harvesting lead to selective genomic differences?; and (4) Do demographic models support increased recruitment at the leading edge during climate anomalies, and increased abundance in areas with harvesting protection? These questions address the complexity of selection forces acting on a species, in hopes that we can better identify and conserve populations that are pre‐adapted to the multitude of threats that species experience. Characterizing these eco‐evolutionary dynamics can inform the conservation management of *L. gigantea* and other key coastal species under rapid climate change and the variability of future human impacts.

## 2 Methods

### 2.1 Molecular Data and Bioinformatics

**FIGURE 2:**
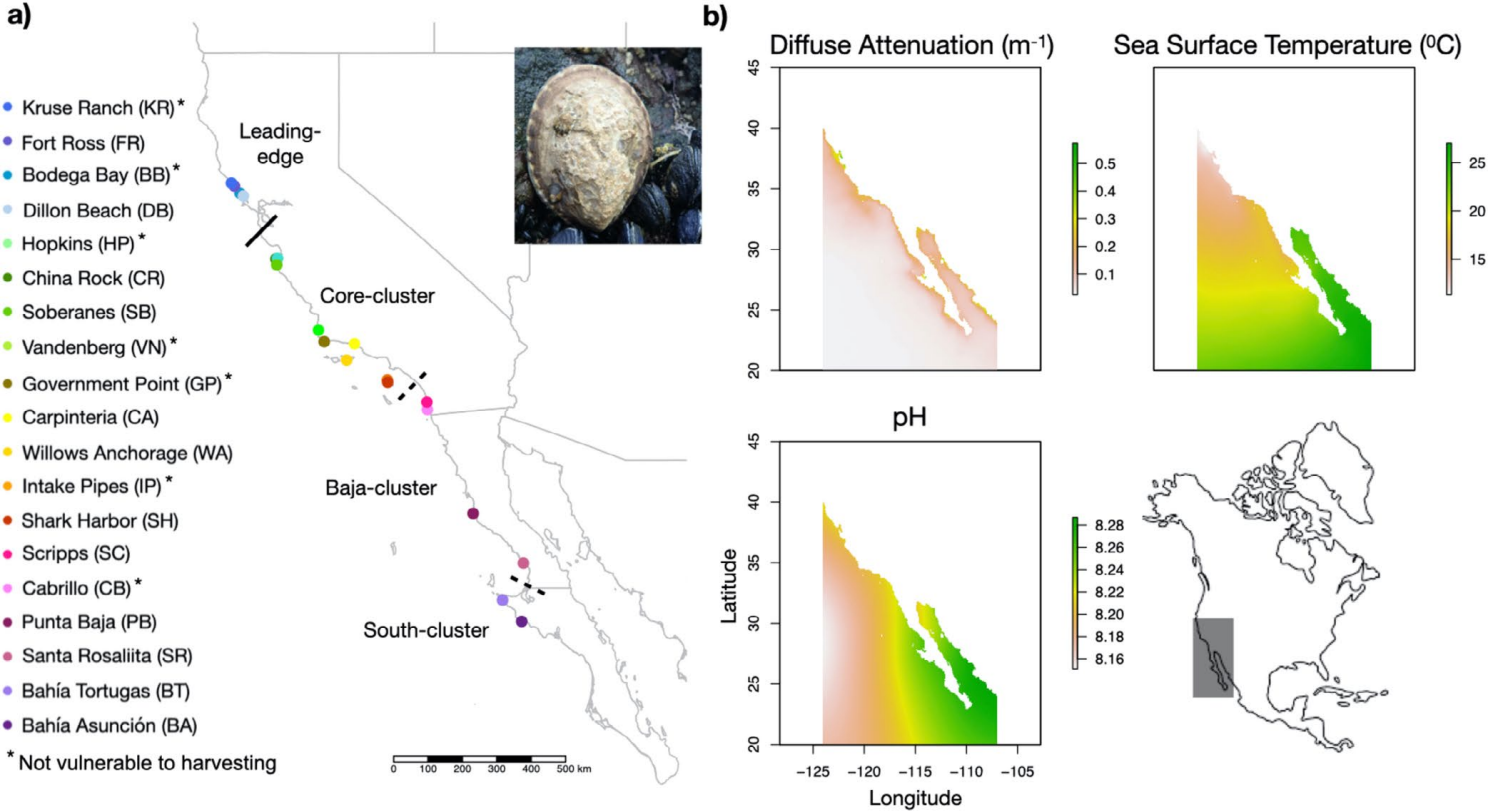
Map of the sample sites for the genomic analyses of *Lottia gigantea* along the Pacific coast of North America (a) with the range of expansion (Leading‐edge) designated by the solid line, as well as the phylogeographic clusters identified by Nielsen et al. (2024) separated by the dashed lines. Sites with asterisks are classified as being not vulnerable to harvesting (vulnerability taken from Sagarin et al. 2007 when available; otherwise classified as vulnerable if outside a protected area and not vulnerable to harvesting within a protected area). Maps displaying the variation in the three environmental predictor variables are shown (b; Assis et al. 2018).

No prior genomic investigations of selection in response to spatio‐temporal environmental variation exist for *L. gigantea* . Our previous phylogeographic study suggests that the species displays low levels of population structure, with breaks in southern California and the Baja California Peninsula (Figure 2a; Nielsen et al. 2024). Population assignment tests and larval simulation models suggest that the recent recruitment event mediated by marine heatwaves originated from the core of the species' distribution, driven by strong northward‐flowing currents (Nielsen et al. 2024). We used the same molecular data from Nielsen et al. (2024), which included 19 sample sites spanning most of the *L. gigantea* distribution (Figure 2a, Table S1: Data S1; SRA BioProject accession number: PRJNA1075458), to test for genomic signals of selection in response to spatio‐temporal environmental variation. Most sites had 30 individuals sampled (although the four Mexico sites had sample numbers ranging from 19 to 29; Table S1: Data S1), representing 10 individuals per size class: small (10–25 mm), medium (30–40 mm), and large (> 40 mm) shell length. There was minimal influence of sample size on genomic variation inferences, shown by a PCA with all sites sub‐sampled to the lowest sample size of 19 individuals, which had the same pattern as that including all individuals (Figure S1: Data S1).

Tissue collection, storage, DNA extraction, and library preparation protocols are provided in Nielsen et al. (2024). To briefly summarize the methods in our previous study, we employed a low‐coverage, whole‐genome sequencing (lcWGS), followed by standard lcWGS quality control filtering and mapping steps (Therkildsen and Palumbi 2017), and SNPs were called from genotype likelihoods using ANGSD (see Nielsen et al. 2024 for full details). We ran the ‐doMAF command in ANGSD with SNPs previously polarized as a major or minor allele (using ‐sites and –doMajorMinor 3) to estimate per‐site minor allele frequencies (MAFs). We called SNPs that were only present in at least 50% of the total individuals and a coverage of below three times the number of individuals across all populations (Lou et al. 2021). SNPs were linkage disequilibrium (LD)‐filtered with plink, leading to a final panel total of 703,925 SNPs.

### 2.2 Selection Across Long‐Standing Environmental Gradients

We used genotype‐environment associations (GEAs) to identify signals of selection across range‐wide environmental gradients, and to understand how the leading‐edge (four northernmost sites) and trailing‐edge populations (the four southernmost sites) may be differentially adapted to long‐standing climatic variables. A total of 16 environmental variables were considered as predictors in the GEAs (see File S1). Oceanographic variables from 2000 to 2014 were downloaded from Bio‐Oracle v2.1 (Assis et al. 2018). Environmental measures, at a resolution of 5 arcmin, were extracted per site using the sdmpredictors (Bosch et al. 2017) and raster (Hijmans et al. 2015) R packages. This full set of environmental variables was filtered to account for collinearity, and we removed those with a Pearson's *R* > 0.7 and variance inflation factor (VIF) > 10. Due to their importance in marine molluscs biology (Bosch et al. 2018), three predictor variables were retained after accounting for collinearity: mean sea surface temperature (SST), diffuse attenuation (DA; a measure of water clarity and turbidity), and pH. Temperature is one of the most important external variables driving physiology and behavior in marine molluscs (Dong et al. 2022) and can influence the susceptibility of *L. gigantea* to predation (Pound 2017) and desiccation (Miller, Harley, and Denny 2009). Diffuse attenuation and its relation to processes/patterns such as downwelling, freshwater discharge, and primary productivity (Hochberg et al. 2020; Simon and Shanmugam 2013) can be used as a proxy for understanding how variables such as near‐shore productivity affect larval transport and survival (Fiksen et al. 2002; Hovel and Morgan 1999). As ocean acidification likely alters *L. gigantea* sensitivity to thermal stress and shell corrosion (Gazeau et al. 2013), with our surveys of northernmost populations anecdotally showing more eroded shells, we included pH as one of the predictor variables in the GEAs.

Isolation‐by‐environment (IBE) tests were conducted to assess the relationship between genomic and environmental differentiation. We used the same three environmental variables in the IBE as in the GEAs (SST, diffuse attenuation, and pH). We assessed isolation‐by‐environment with partial Mantel tests with the ecodist R package (Goslee and Urban 2007) using 1000 permutations. Model significance was assessed with *q*‐values generated by the *q*‐value R package, using a false discovery rate (FDR) of 0.05. We found no significant isolation‐by‐distance (IBD) in our phylogeographic study (Nielsen et al. 2024); thus, IBE analyses are unlikely to be confounded by IBD.

Range‐wide GEA tests were conducted on per‐site SNP allele frequencies using three independent programs/models: BayPass (Gautier 2015), latent factor mixed models (LFMM2; Caye et al. 2019), and Redundancy Analysis (RDA). We ran the auxiliary model of BayPass v2.2, which computes a Bayes factor (BF) to classify the association between SNP frequency and an environmental variable for each locus while accounting for multiple tests (Gautier 2015). We scaled the environmental variables in the model with ‐scalecov, and population structure was controlled for with the inclusion of a covariance matrix created in the core BayPass model. Outliers were identified as those with a log10 Bayes factor (db) > 20 (Jefferys 1961), after values were averaged across separate model runs. To determine the number of clusters, *K*, to constrain the LFMM, we ran the snmf function of the LEA R package (Frichot and François 2015), testing *K* from 1 to 10. LFMM outliers were then identified using the lfmm2 R package, using an FDR < 0.05, manually adjusting the *p*‐values based on the genomic inflation factor (GIF) per environmental variable. RDAs were run using the rda function of the vegan R package, with scale = T (Oksanen et al. 2013). Significance of the model, predictor variables, and RDA axes was determined using an ANOVA with 1000 permutations. Outlier loci were identified as those with loading scores +/− three standard deviations from the mean loading for either of the first two axes (Forester et al. 2017).

Loci selected by at least two of the three GEA models were partitioned into the ‘range‐wide outlier’ dataset. To assess geographic variation at these loci, we ran a principal components analysis (PCA) on the range‐wide outliers. The PCA was created with pcangsd (Meisner et al. 2021) using a .beagle input file created from ANGSD ‘‐doGlf 2’ with the ‘‐sites’ pertaining to the list of outliers. We performed an additional RDA solely on the range‐wide outlier dataset, as this allows for better identification of the environmental associations from the ‘adaptively enriched space’ of the outlier SNP frequencies (Capblancq et al. 2020). To assess range‐wide patterns of adaptive capacity, we calculated the standing genetic variation (SGV; Chhatre et al. 2019) and population adaptive index (PAI; Bonin et al. 2007) from the range‐wide outlier dataset. Standing genetic variation is a measure of within‐population diversity (e.g., alpha diversity) and is the mean of the allele frequency variances (*pq* = p*(1 − *p*)), while PAI is a measure of between‐population diversity (e.g., beta diversity) and is calculated as the absolute difference in the allele frequencies of each SNP in a specific population and the mean allele frequency of that SNP across all populations.

### 2.3 Selection Associated With a Recent Range Expansion

To assess selection specific to the range expansion of the leading‐edge populations, we ran additional outlier detection analyses. These outlier detection analyses were run on the full LD‐pruned SNP panel and all populations. We first ran the BayPass contrast statistics (C2) model, which is the core model based on the scaled covariance matrix of population allele frequencies (*Ω*), but assesses differences between two predefined groupings (range core versus leading‐edge). We contrasted the leading‐edge sites to those from the core cluster (Figure 2), using default model parameters with 30 pilot runs. Outliers were identified as those with an FDR < 0.05, after converting *p*‐values from the summary_contrast.out file to FDR using the *p*.adjust function of the R stats package. We also performed outlier scans using the population branch statistic (PBS; Yelmen et al. 2021). PBS identifies significant differences in allele frequencies between two closely related populations, using a third as an outgroup (Burri 2017), and can detect recent selection under a neutral demographic model (Yi et al. 2010). It ranks loci based on their PBS score, which represents the deviation in genomic differentiation of each locus from the expected differentiation given all loci. Here, we used the program PBScan (Hämälä and Savolainen 2019), estimating the genotype likelihoods with ANGSD. We compared allele frequencies of the leading‐edge and core cluster, using the Baja cluster as an outgroup (Figure 2). PBScan was run with default parameters, and outliers were identified as those with scores above the 99.95th percentile (Rougemont et al. 2020). Any loci that were selected by both the BayPass C2 model and PBS were classified as ‘leading‐edge outliers’. Subsequent PCAs and RDAs were run on the subset of leading‐edge outliers following the same procedure as above.

### 2.4 Genomic Signals of Harvesting Vulnerability

To assess the influence of size‐selective harvesting on the genomic composition of *L. gigantea* , we ran pairwise comparative analyses on sites characterized as vulnerable to harvesting or not, with six site‐pairs across the species' California distribution (Figure 2). Only the California sites were included in the human harvesting analyses, as harvesting vulnerability data were unavailable for Mexico sites. Furthermore, to limit environmental differences between site‐pairs, we chose pairs of sites that were most or least vulnerable to harvesting in close geographic proximity. To classify sample sites as either vulnerable to harvesting or not, we used the vulnerability categories defined by Sagarin et al. (2007): sites with a vulnerability category of 1 (most vulnerable) were here classified as vulnerable to harvesting, and sites with categories of 2 or 3 (little to no expected collection and well‐enforced restrictions against collections) were classified as not vulnerable to harvesting. For sample sites whose vulnerability categories were not included in Sagarin et al. (2007), we classified sites within a protected area (such as a Marine Protected Area, or MPA) as not vulnerable to harvesting and those that are unprotected as vulnerable to harvesting. We compared a measure of genomic diversity, expected heterozygosity (*H*_e_), between sites either vulnerable to harvesting or not. *H*_e_ was estimated for all individuals, as well as solely large individuals (to account for size‐selective harvesting of larger individuals). We calculated *H*_e_ from genotype likelihoods from site‐frequency selections generated from ANGSD, using the following scripts: git@github.com:sbarfield/yap_ahyacinthus‐.git/heterozygosity_beagle.r.

We used multiple analyses to investigate potential signals of selection associated with harvesting. We ran an additional RDA on the allele frequencies from California sampling sites only, including the same three environmental variables as above, but additionally including harvesting vulnerability as a binary predictor variable. To identify loci that might be under selection from harvesting, we ran the following outlier detection approaches: the BayPass C2 model as stated before, but with groups consisting of sites either vulnerable to harvesting or not, and a Cochran–Mantel–Haenszel (CMH) chi‐squared test (Zhang and Boos 1997). We compared per‐site allele frequencies between the two harvesting vulnerability groups with the ‘cmh.test’ command of the poolseq R package, and adjusted the *p*‐values for multiple testing with the *q*value R package. Outlier SNPs were identified as those with an FDR < 0.05 (Benjamini and Hochberg 1995). Outliers chosen by either of these methods created the ‘harvesting outlier’ dataset and were used to create additional PCAs of the variation within these harvesting‐associated loci.

### 2.5 Gene Ontology

We used Gene Ontology (GO) categories to characterize the functionality of genes associated with the identified outlier SNPs. We used LD‐annot v.0.4 (Prunier et al. 2019) to identify genes in linkage disequilibrium with the outlier SNP datasets, using an *r* ^2^ threshold of 0.9. The gene IDs identified from LD‐annot were then used to create a gene list using the ‘lgigantea_eg_gene’ dataset within the biomaRt R package. This gene list was input into TopGO v.2.40.0 (Alexa and Rahnenführer 2009) to assess whether the outlier‐linked genes were enriched for specific gene ontology terms. We used Fisher's exact test to assess the significance of the enriched gene ontology terms, with a significance threshold from the row *p*‐values (per GO term) of *p* < 0.05 from the getSigGroups function (Alexa and Rahnenführer 2009).

### 2.6 Assessing Impacts of Climatic Anomalies and Marine Protection on Demography

As this study assesses the influence of recent climatic and anthropogenic events (i.e., heatwave‐driven range expansion and size‐selective harvesting) on the genomic composition of *L. gigantea* , we explored the demographic changes caused by these events. We used hierarchical Generalized Additive Models (HGAMs) to ‘ground truth’ the expected demographic changes that occur in response to marine heatwave anomalies and potential harvesting pressure. We ran HGAMs instead of non‐hierarchical GAMs to describe differences in population trends between size classes. GAMs are useful tools for spatio‐temporal modeling as they can handle nonlinear relationships between a response variable (such as species abundance) and multiple predictor variables (such as environmental variables; Wood 2024). GAMs are similar to generalized linear models but incorporate nonparametric smoothing functions, termed “smooths”, which minimize residual error and overfitting through the use of penalty matrices (Wood 2024). Hierarchical GAMs expand on the GAM framework of smoothed functional relationships between predictor and response variables by allowing these relationships to vary between groups (Pedersen et al. 2019). Within our models, we used thin plate splines, which penalize changes in the derivative(s) of a function. As we were interested in assessing interactions between predictor variables, the models also include tensor product smoothers, which are analogous to interaction terms in mixed effect models (Pedersen et al. 2019; Wood 2024).

We obtained count data from the Multi‐Agency Rocky Intertidal Network (MARINe) monitoring program (marine.ucsc.edu, 2022). The surveys targeted *L. gigantea* , counting all individuals within a 1 m radius fixed circular plot to obtain annual counts and size measurements. Sixty‐five sites between ~27.8° N and 38.6° N latitude were sampled from 1995 to 2020 (Figure S2: Data S1). These sites were classified as either within an MPA or unprotected, which we used as a proxy for harvesting vulnerability. We extracted the number of plots sampled for each observation date and the total number of individuals counted in all plots per size bin (binned to the nearest millimeter). We summed raw counts for the following size bins per site: small (< 26 mm), medium (26–40 mm), and large (> 40 mm).

We ran two HGAMs, the first of which assessed whether abundance trends differed between MPAs and unprotected sites, as well as between the different size classes, and whether marine protection differentially affected the three size classes. This model included a thin plate smooth year, as well as a smooth of year by size or protection, and a smooth of year by an interaction between size class and protection (see Data S1 for code). The second model tested the effect of latitude on abundance trends over time, and whether this differed by size class. This model included smooths for year and latitude and latitude by size, as well as tensor smooths for year plus latitude and year plus latitude by size (Data S1). We used a negative binomial distribution to account for overdispersion in the species count data (Stoklosa et al. 2022), and used restricted maximum likelihood (REML) to fit the smoothing parameters. Models also included an ‘offset’ term to account for different numbers of plots sampled per site. We increased model ‘wiggliness’ by testing the number of knots (*K* = 5, 10, 15, 20) for the smoothing function. *K* = 20 was selected for both as it captured variation in the smoother and effective degrees of freedom were well below *K* (Pedersen et al. 2019). As the Akaike's information criterion is not a robust measure of model fit in HGAMs (Pedersen et al. 2019), we assessed models based on their REML scores as well as root‐mean‐squared residuals (RMSE). HGAMs were run using the ‘gam’ function of the mgcv package (Wood 2000).

## 3 Results

### 3.1 Selection Across Long‐Standing Environmental Gradients

**FIGURE 3:**
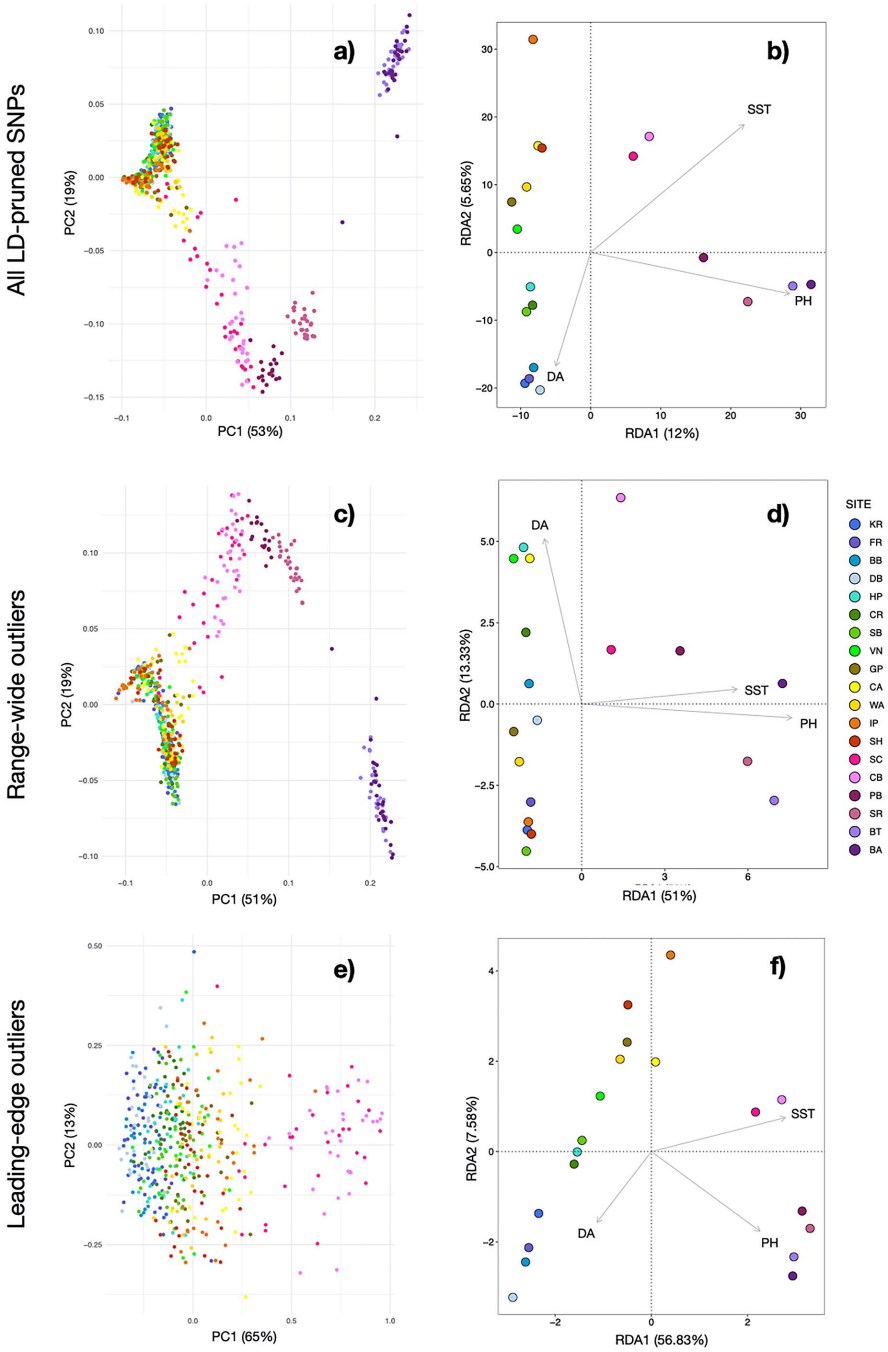
Principal Components Analysis (PCA) based on the linkage‐disequilibrium (LD) SNP *Lottia gigantea* dataset (redrawn from Nielsen et al. 2024) shows predominantly neutral genetic variation (a). A redundancy analysis (RDA) on the LD‐pruned SNP dataset shows genomic variation in relation to sea‐surface temperatures (SST), diffuse attenuation (DA; a measure of water clarity and turbidity), and pH (b). Genomic structuring of the 2189 range‐wide outliers identified by at least two of the three genotype‐environment association tests is shown by a Principal Components Analysis (c) and RDA (d). Genomic variation within the leading‐edge outlier SNPs is shown in a PCA (e) and RDA (f) of the 360 total loci within this dataset. Site abbreviations and locations are listed in Figure 2.

We identified genomic signals of selection associated with range‐wide environmental gradients. The LD‐filtered panel of 703,925 SNPs was used for isolation‐by‐environment and genotype‐environment association analyses. The PCA of the full LD‐pruned SNP dataset displayed differentiation mainly within the southern portion of the species range (Figure 3a). Californian populations were differentiated from one another along a sea surface temperature (SST) gradient, and California was differentiated from Mexico along a pH axis (Figure 3b). There was significant isolation‐by‐environment with SST (*r* ^2^ = 0.22, *q*val = 0.026) and pH (*r* ^2^ = 0.69, *q*val = 0.004), but not with diffuse attenuation (*r* ^2^ = −0.18, *q*val = 0.83; SM2 Table S2). The full SNP dataset showed significant variation based on the three environmental variables included in the RDA (*p* = 0.001, adj*R* ^2^ = 0.073, 17.6% variation explained; Figure 3b). Of the three environmental variables, SST and pH were significant predictors of genomic variation in the RDA (Table S3: Data S1). The four Mexico sites could be distinguished from all other populations mainly along the pH axis, whereas the SST axis distinguished the southern California sites (Figure 3b). The central/northern California sites geographically clustered, mainly along the diffuse attenuation axis (Figure 3b). BayPass selected a total of 404 outlier SNPs, compared to the 6663 selected by LFMM and 1232 by RDA (Table S4: Data S1). A total of 2189 SNPs were selected by at least two of the three outlier methods (Table S5: Data S1) and went into the ‘range‐wide outlier’ dataset.

Variation within range‐wide outlier SNPs followed a similar pattern to the full SNP dataset (Figure 3), with the exception that range‐wide outliers no longer showed latitudinal groupings within the core and leading‐edge populations (Figure 3c,d). These core and northern California sites also displayed a single cluster when assessed without the Mexico and southern California sites (Figure S3: Data S1). All three predictor variables were significant drivers of genomic variation within RDA run solely on range‐wide outlier SNPs (Figure 3d, Table S3: Data S1). Similar to the full SNP dataset, the Mexico and two southernmost California sites were differentiated from the remainder of the sites, mainly along the SST and pH axes (Figure 3d). The range‐wide outlier SNPs showed no differentiation specific to the expanded range (the four northernmost sites).

**FIGURE 4:**
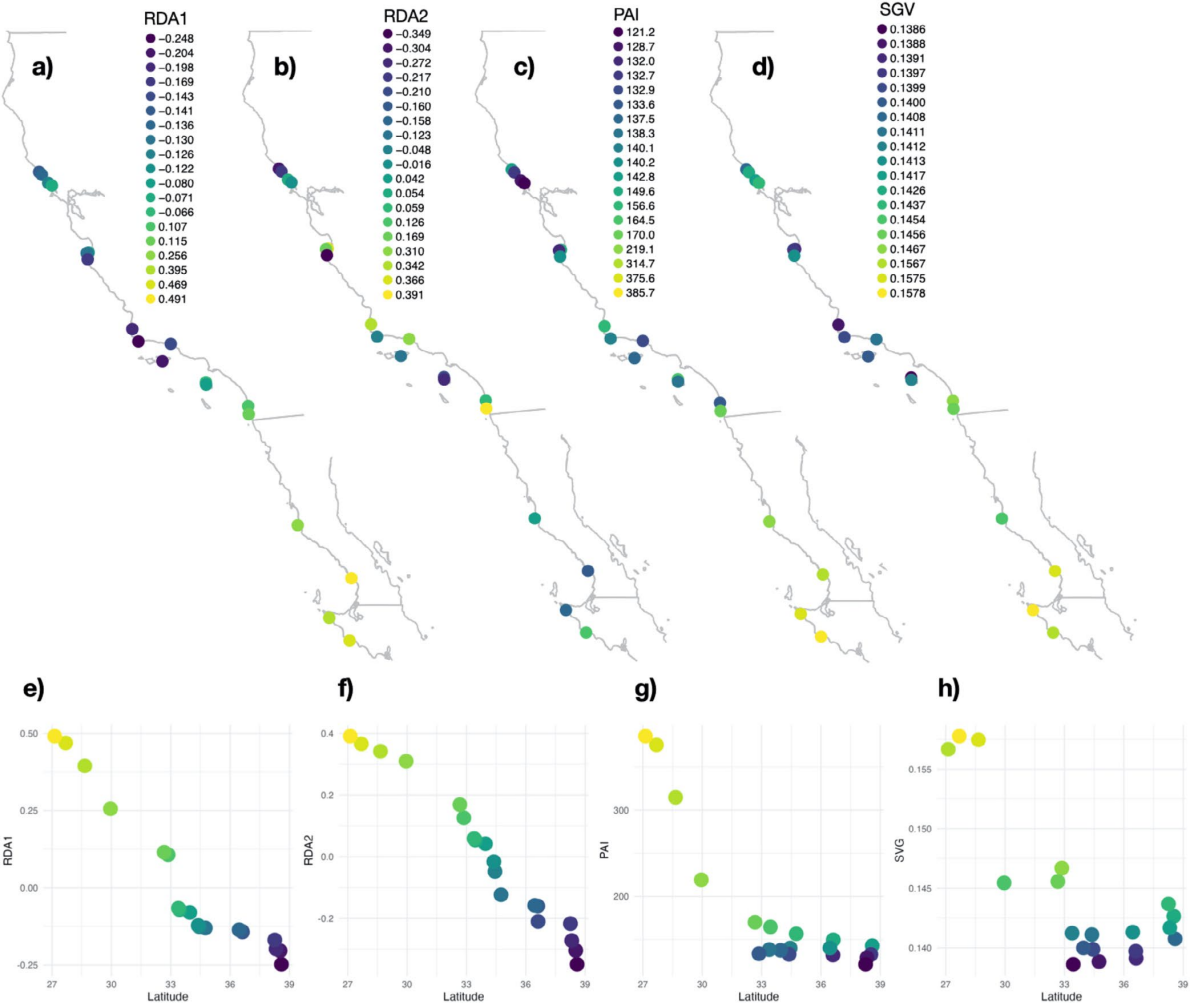
Spatial patterns of adaptive potential across the 19 sample sites based on the range‐wide GEA outliers are shown by maps of RDA1 (a) and RDA2 (b) from the RDA of the outlier SNPs (see Figure 3b), as well as the per‐site population adaptive index (PAI; c) and standing genetic variation (SGV; d) calculated from the outlier SNPs. Values of the following are also plotted against latitude: RDA1 (e), RDA2 (f), PAI (g), and SGV (h).

Metrics of adaptive capacity based on range‐wide outliers mostly (but not exclusively) follow latitudinal patterns. Most of the variation in range‐wide outlier SNPs was associated with SST and pH (RDA1, Figure 3d), while the second axis of variation was mainly associated with diffuse attenuation (RDA2, Figure 3d). Mapping RDA1 captured the latitudinal gradients in SST and pH, with Mexico and central California sites as the most differentiated within this adaptive component (Figure 4a). The spatial pattern of RDA2, which was associated with diffuse attenuation, showed less of a latitudinal gradient and more differentiation within the California core and northern sites (Figure 4b). The southernmost sites had the highest values of both population adaptive index (PAI) and standing genetic variation (SGV) within range‐wide outliers (Figure 4c,d). We found a lower population adaptive index in the expanded range, while adaptive standing genetic variation had the lowest values within the core of the species' range (Figure 4c,d). Values of RDA1 and RDA2 show a gradual cline across latitudes (Figure 4e,f), while values of PAI and SGV show large breaks around 30° and 33° latitudes (Figure 4g,h). Range‐wide outliers identified 101 gene ontology terms, which included broad functions such as enzyme activity, biological adhesion and binding, and cell communication and signaling, among others (Table S6: Data S1).

### 3.2 Selection Associated With a Recent Range Expansion

The leading‐edge SNPs showed genomic differentiation between the expanded range and the range core, which was not captured by the range‐wide outlier SNPs. Of the analyses specifically testing selection associated with the recent range expansion, BayPass C2 identified 8944 outliers and PBS identified 572, with a total of 360 overlapping SNPs between the two. Of these 360 SNPs, 118 were also within the range‐wide outlier dataset. While there was no strong clustering between sites, the leading‐edge outliers showed some differentiation between the expanded range and the rest of the California core sites (Figure 3e). The leading‐edge outlier SNP dataset displayed genomic structuring of the southern California and Mexico sites, differentiated along the SST and pH axes (Figure 3f). However, within this SNP dataset we did see a clear distinction between the expanded range and northern core sites, which was mainly along the diffuse attenuation axis (Figure 3f). Clustering of just the California core and leading‐edge sites (omitting southern California and Mexico) further distinguished the four leading‐edge sites (Figure S4: Data S1). Leading‐edge outliers identified six gene ontology terms, which are distinct from the gene ontology terms of the range‐wide outliers. The gene ontology terms included several RNA‐related functions, such as ncRNA metabolic process, RNA binding, and catalytic activity, acting on RNA, as well as other processes such as iron ion binding and transcription coregulator activity (Table S7: Data S1).

### 3.3 Genomic Signals Associated With Harvesting Vulnerability

**FIGURE 5:**
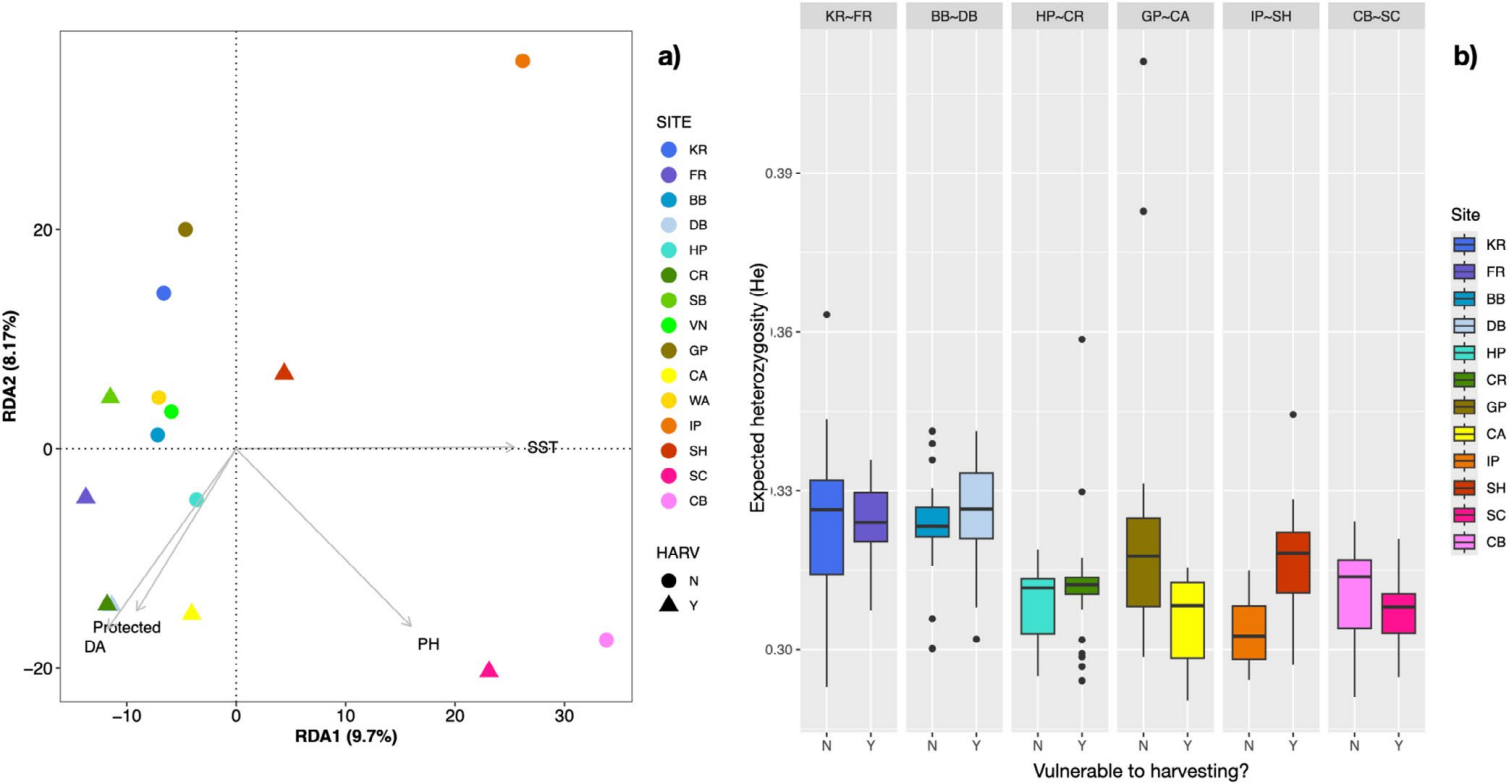
Genomic signals of harvesting pressure are shown in a Redundancy Analysis (RDA) of outliers identified by allele frequency differences between paired California sites either vulnerable to harvesting or not (a). The RDA shows genomic variation within the SNP dataset in relation to the sea‐surface temperatures (SST), diffuse attenuation (DA; a measure of water clarity and turbidity), pH, and protection level, with colors representing site latitude and shapes indicating whether it is vulnerable to harvesting (Y = triangles) or not (*N* = circles). Genomic diversity is also compared between pairs of protected versus unprotected California sites (b), shown by boxplots of expected heterozygosity (*H*_*e*_) values. Site abbreviations and locations are listed in Figure 2.

We found little evidence of harvesting vulnerability affecting genetic variation in *L. gigantea* . When comparing sites either vulnerable to harvesting or not, the BayPass C2 model identified 207 outliers, and the CMH test identified zero outliers. Of the 207 outliers identified by BayPass, 131 overlapped with range‐wide GEA outliers, and 41 overlapped with leading‐edge outliers. Clustering of harvesting outliers showed no distinction between sites vulnerable to harvesting or not (Figures 5a and S5: Data S1). Harvesting level was not a significant predictor in the RDA (i.e., did not cause significant clustering within the model; Figure 5a). We also found little evidence that sites not vulnerable to harvesting have higher genomic diversity (Figure 5b), even when solely including large individuals (Figure S6: Data S1). The only significant gene ontology terms associated with the harvesting outliers were those associated with biological processing and mainly consisted of cellular signaling and response to stimulus functions (Table S8: Data S1).

### 3.4 Abundance Trends by Size, Latitude, and Marine Protection Level

**FIGURE 6:**
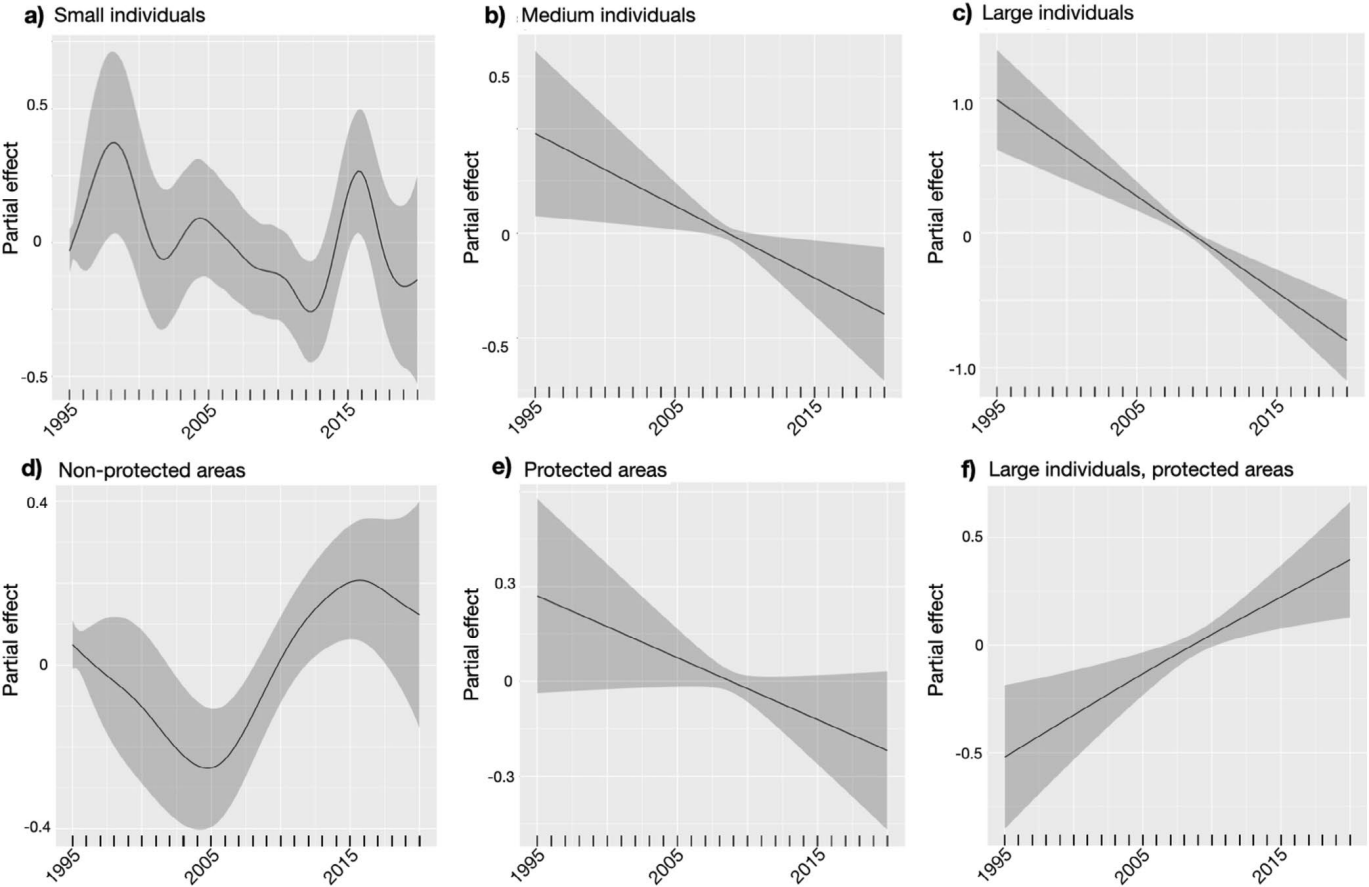
Hierarchical Generalized Additive Model (HGAM) plots showing the partial effect of the selected variables on the abundance trends of *Lottia gigantea* over time from 1995 to 2020. Effects are shown for size classes with small (< 26 mm; a), medium (26–40 mm; b), and large (> 40 mm; c). Trends are also shown for non‐marine protected areas (d), marine protected sites (e) as well as the interaction between protection and size (protected sites and large individuals; f). Shaded ribbons indicate 95% confidence intervals.

Counts of all size classes of *L. gigantea* varied over the 25 years with peaks in abundance around the 1997–1998 El Niño and the 2014–2016 marine heatwave anomalies (Figure S7: Data S1). Size‐specific models showed that the abundance of medium and large individuals decreased over time, but the abundance of small individuals fluctuated, with peaks around 1998 and 2016 (Figure 6a–c). Predicted abundance for all size classes combined was generally higher south of ~36.5° N (Monterey, California; Figure S7: Data S1). There was no significant trend in the abundance of medium individuals across latitude. The abundance of large individuals varied across latitude with peaks around 34.5° N and 37° N latitude, while the abundance of small individuals peaked around 33.5° N with a decline starting around 37° N (Figure S8: Data S1). Modeling the interaction of year and latitude for all individuals showed an increase in abundance following 2015, mainly within the northern part of the species' range (Figure S7c: Data S1). The MPA sites showed a negative trend over time, while the unprotected sites showed a non‐linear trajectory, decreasing until ~2005, then increasing until ~2015, and decreasing since (Figure 6d,e). There was no significant difference between abundances in protected or unprotected sites for all size classes combined. The only significant interaction between size and protection was for large individuals, which had an increasing trend in protected sites, and which differed from the overall negative trend of declining numbers of large individuals over time (Figure 6c,f).

## 4 Discussion

Understanding how both climatic trends and short‐term anomalies shape the adaptive potential of populations is pivotal to conserving intraspecific variation under global change (Andrello et al. 2022; Sgrò et al. 2011). Rising temperatures and ocean acidification associated with global change, as well as human exploitation, are leading to novel eco‐evolutionary responses within marine systems (Harley et al. 2006; Poloczanska et al. 2013). Here, we investigate how long‐standing environmental gradients, recent climatic anomalies, and size‐selective harvesting shape the adaptive landscape of *L. gigantea* across most of its geographic range. Largely, each of these selection forces has distinct genomic imprints. We find no genomic signal of harvesting, despite notable demographic effects. Across the entire range, genomic signals of selection are primarily associated with sea surface temperature and pH gradients. While these “range‐wide outliers” do not distinguish range‐core from leading‐edge populations, a distinct set of SNPs (the “leading‐edge outliers”) shows signals of selection in the expanded range. This pattern suggests that genomic variation from long‐term selection due to environmental variation across a species' range is at least in part distinct from that associated with short‐term environmental change. This study highlights the importance of assessing adaptive potential over multiple spatial and temporal scales, and how traditional gene–environment association tests might not capture selection from recent climatic anomalies.

### 4.1 Range‐Wide and Leading‐Edge Selection

Genomic variation suggests potential adaptation to environmental gradients across the species' range. This finding supports a growing literature showing that selection can be prominent even in marine species with high gene flow and long dispersal potential (Hellberg 2009; Sanford and Kelly 2011; Tigano and Friesen 2016). This pattern of selection against a backdrop of genetic mixing is evident in the contrast between geographic signals revealed by neutral processes, which act on the whole genome, and selective processes, which act on subsets of the genome. For example, Coscia et al. (2019) found for the common cockle *Cerastoderma edule* that neutral genetic divergence was more strongly associated with geographic distance between sites, and that outlier divergence was more strongly driven by differences in SSTs. Similarly, our results display geographic groupings in neutral SNPs, but not in range‐wide outliers (Figure 3b,d).

The range‐wide SNPs show the strongest genomic signals of selection associated with pH and SST gradients, with pH primarily associated with the differentiation of the Mexico sites, and SST with the southern California sites. Sea surface temperature is a common driver of selection in marine species (Benestan et al. 2016; Cayuela et al. 2020; Hu and Dong 2022; Selkoe et al. 2016; Stanley et al. 2018), and likely also captures selective pressures of other correlated environmental variables, such as coastal upwelling and nutrient availability. Temperature influences most biological processes and is a strong determinant of marine biodiversity (Antão et al. 2020), while pH affects the growth and calcification of many marine gastropods (Kroeker et al. 2011; Ries et al. 2009). Additionally, low pH can reduce anti‐predator responses and alter foraging rates of marine snails (Jellison et al. 2022).

In comparison to SST and pH, diffuse attenuation is mainly associated with the differentiation of the central and northern California sites. Diffuse attenuation reflects water turbidity and primary productivity in the water column, both of which affect the growth of the microalgae that *L. gigantea* consumes (Bosnian and Hockey 1988). Temperature, pH, and turbidity also influence larval development and settlement success (Raventos et al. 2021; Robitzch et al. 2016), and may contribute to patterns of genetic differentiation of marine species (Cayuela et al. 2020; Dallaire et al. 2021; Torrado et al. 2020).

Genotype‐environmental association (GEA) studies have previously identified selection at the leading edge following range expansions. For example, leading‐edge populations show divergent genotype‐climate associations with temperature and precipitation in the range‐expanding damselfly *Ischnura elegans* (Dudaniec et al. 2018) and the invasive starling *Sturnus vulgaris* (Hofmeister et al. 2021). However, GEAs have yet to assess local adaptation of a range‐expansion event in any marine species. Within the range‐wide environmentally associated SNPs we did not observe a strong signal of local adaptation of the leading‐edge populations to the environmental predictors (and associated variables; Figure 3c,d). This finding is not surprising as the environmental differentiation within the northern half of the species' range is not as pronounced as within the southern half (Figure 1), and the short timescale of the expansion event might not allow for this environmental selection to be detectable in the GEAs. Previous work on *L. gigantea* indicates that the range boundary at the leading edge is more likely due to limited recruitment rather than the availability of suitable habitat (Fenberg and Rivadeneira 2011) and anomalously strong northward‐flowing currents and warmer water temperatures during the 2014–2016 marine heatwaves allowed for above‐average recruitment (Nielsen et al. 2024; Sanford et al. 2019). Similarly, Gilman (2006) found that limitations on recruitment, rather than adult performance, led to decreases in abundance at the poleward edge of a related intertidal gastropod, *Lottia scabra* .

There are some limits to using genotype‐association analyses to characterize outlier SNPs potentially under selection from range expansions. First, these analyses can yield false positives/negatives, caused by variation in chromosome lengths (Salmón et al. 2021), recombination rates (Rougemont et al. 2020), and polygenic traits due to small frequency shifts across multiple alleles (Yeaman 2015). Second, drift and allele surfing could lead to neutral variation in the expanded range, which is difficult to differentiate from adaptive variation. However, there is no evidence that the four northern sites exhibit reduced genetic diversity or signatures of allele surfing (Nielsen et al. 2024), which reduces the likelihood of false positive outliers associated with the expanded range sites (Zhao et al. 2020). Finally, if the expansion event is recent, not enough time may have passed for the demographic changes to affect genomic variation.

*Ad‐hoc* investigations of SNPs differentiating the core from the expanded range revealed candidate SNPs that may reflect selection during range expansion (“leading‐edge” outlier SNPs). Compared to the range‐wide outliers, the leading‐edge outliers differ between the four expanded range sites and the rest of the species' range. Thus, leading‐edge populations may harbor selective signals that are distinct from background long‐standing environmental variation, consistent with selection on larval or juvenile stages during the expansion event (Kelly and Griffiths 2021; Searcy and Sponaugle 2001). The gene ontology terms differ between these two outlier datasets, with the range‐wide outliers relating to functions such as enzyme activity, biological adhesion and binding, and cell communication and signaling. In contrast, the leading‐edge outliers relate to ncRNA metabolic process, RNA binding, and catalytic activity acting on RNA, among others. Interestingly, the leading‐edge gene ontology terms are predominantly associated with RNA functions, which could indicate plastic responses of leading‐edge populations to the range expansion event (DeBiasse and Kelly 2016; Kilvitis et al. 2017), warranting future work with transcriptomics.

We are also currently studying phenotypic and behavioral differences between core and expanded range populations of *L. gigantea* , which could improve our understanding of the selective regime across these regions. Evolutionary theory suggests that trade‐offs should occur during range shifts, with dispersal and reproduction selected for in the leading edge, but at the expense of other traits such as competitive ability (Burton et al. 2010). Such analyses linking traits such as developmental rate and larval duration to genomic variation are pivotal to advancing our understanding of the full breadth of the eco‐evolutionary dynamics associated with marine range expansions.

### 4.2 Demographic Trends and Evolutionary Responses to Harvesting

The HGAMs support our expectation of marine heatwaves leading to demographic shifts within leading‐edge sites (specifically increases in small individuals reflecting increasing recruitment) and offer some support for MPAs being less vulnerable to size‐selective harvesting (shown by the significant positive interaction between protection and large individual abundances). We found an increase in the abundance of small individuals in association with the 1997–1998 El Niño and the 2014–2016 marine heatwaves (particularly in the north), further highlighting the influence of these short‐term climatic anomalies on the demography of the species. The abundance of small individuals fluctuated over time across all sites, suggesting interannual variation in successful recruitment. Previous surveys before the 2014–2016 marine heatwaves also showed a low number of juveniles towards the northern edge of the *L. gigantea* range (zero juveniles present north of ~38° N), suggesting this region might be recruitment limited and demographically unstable (Fenberg and Rivadeneira 2011). Similar to owl limpets, there was a large episodic increase in barnacle recruitment across 750 km of California's coast in association with the 1997–1998 El Niño (Connolly and Roughgarden 1999). Additionally, the spawning success of > 20 intertidal marine invertebrates was orders of magnitude lower at Coos Bay, Oregon, during the 2015–2016 marine heatwaves (Shanks et al. 2020), highlighting how these extreme climatic events can influence spawning and increase interannual variability in recruitment. As these extreme climate events become more frequent (Oliver et al. 2018), it is important that we predict their demographic and evolutionary consequences.

Being within an MPA had no significant effect on abundance for all size classes combined, but there is a significant interaction between protection status and the abundance of large individuals. Compared to the overall trend of large individuals decreasing in abundance over time, large individuals within MPAs show an increasing trend. This trend supports previous work suggesting that protected areas are effective management tools to support species that are potentially subjected to size‐selective harvesting (Fenberg and Roy 2008; Fernández‐Chacón et al. 2020; Roy et al. 2003). However, our findings show an overall decreasing trend in *L. gigantea* counts across all size classes within protected sites (Figure 6e). This negative abundance trend within protected areas could be owing to several factors, such as MPAs not being well‐enforced, or that environmental variables are stronger drivers of abundance and create this negative trend. Negative human impacts can occur within protected areas, and as visitation to MPAs in southern California increased in the past two decades, these protected sites might experience increased anthropogenic pressures such as trampling (Lucas and Smith 2016). Level of visitation (and associated damages via trampling and handling) might be a better predictor of *L. gigantea* population densities than protection banning harvesting, which was found to be the case for California mussel *Mytilus californianus* ) populations (Smith et al. 2008).

Despite our demographic models indicating that protected areas increase the number of large *L. gigantea* individuals, we found no significant differences in the genomic composition of populations classified as vulnerable to harvesting or not within California. Examples of exploited marine invertebrates harboring lower genetic diversity due to harvesting are rare (De Croos and Pálsson 2012), possibly due to high effective population sizes and high gene flow in these taxa. In fact, multiple studies have found no difference, or even higher genetic diversity within unprotected or exploited populations of marine species (Arnaldi et al. 2018; Bell and Okamura 2005; Miller, Maynard, and Mundy 2009; Yorisue et al. 2020). For example, genetic diversity for three exploited marine species did not differ between protected and unprotected areas across a network of eight MPAs within the Mediterranean Sea (Benestan et al. 2023).

The lack of genetic differentiation across populations of *L. gigantea* subject to differences in harvesting vulnerability could be due to spillover from non‐harvested to harvested areas, as this species shows high levels of population connectivity (and pelagic larval duration of ~4–21 days; Fenberg et al. 2010; Nielsen et al. 2024; Sanford et al., in prep). Fenberg et al. (2010) also suggest that the phenotypic differences between exploited and protected populations of *L. gigantea* could be a plastic rather than a genetic response to harvesting pressure. We did find weak evidence of selection via harvesting, as the BayPass outlier C2 model identified 207 SNPs associated with harvesting vulnerability. However, clustering analyses of these outliers showed no distinction between harvesting vulnerability categories (Figure S5: DataS1), suggesting there is no strong harvesting variation within these SNPs. Ultimately, our study is limited in drawing conclusions on the genomic effects of harvesting, as we lack data on harvesting pressure. Vulnerability to harvesting classifications from Sagarin et al. (2007) and MPA status are only proxies for harvesting, and this work highlights how the data gap on intertidal harvesting limits our understanding of anthropogenic threats to rocky shore species.

### 4.3 Conservation Implications

Understanding how selection imposed by variation in environmental and anthropogenic pressures shapes genomic differentiation is essential to predicting intraspecific vulnerability and prioritizing ecological and evolutionary units for conservation (Benestan 2019; Capblancq et al. 2020; Funk et al. 2019). We found distinct genomic selective signals from potential pressures exerted by long‐standing environmental gradients versus climatic anomalies, yet no influence of size‐selective harvesting on genomic variation. Our results also suggest that the range edges contain populations of conservation importance, with the trailing edge harboring high levels of alleles adapted to range‐wide environmental pressures and the leading edge harboring alleles that may have an adaptive advantage if and when the northern range expands. Our demographic models indicate a positive impact of MPAs on the abundance of large individuals, suggesting that MPA designation is an effective conservation strategy to limit size‐selective harvesting. However, the models also showed decreasing *L. gigantea* abundance over time across all size classes within MPAs, which means that to best safeguard the adaptive potential of the species, greater MPA enforcement or other regulations may be needed.

While the trailing‐edge populations have high neutral (Nielsen et al. 2024) and adaptive genomic diversity increasing their adaptive potential, they are also more likely to be living at the edge of their upper physiological limits, making them more vulnerable to increasing thermal stress than poleward leading‐edge populations (Gilbert et al. 2020; Hoffmann and Sgrò 2011; Pörtner and Gutt 2016). In the trailing‐edge of the species' range, there is lower dispersal and more fragmented habitat (Fenberg and Rivadeneira 2011), which increases the likelihood of local adaptation, while also decreasing the likelihood of adaptive plasticity (Usui et al. 2023). The higher genetic diversity and environmentally associated alleles within the trailing‐edge increase the capacity for these populations to persist under changing climatic conditions. However, these trailing‐edge populations are at risk of ocean warming increasing isolation between habitat patches (via shorter pelagic larval durations at warmer temperatures; Kendall et al. 2013), and harvesting potentially further reducing effective population size. These processes may operate synergistically to cause the rapid collapse of these populations (Harley and Rogers‐Bennett 2004). Protecting the range edges of *L. gigantea* from harvesting may be an appropriate strategy to safeguard the unique warm‐adapted alleles of the trailing‐edge populations offering some buffer against any additive effects of climate change and harvesting pressure (Harley and Rogers‐Bennett 2004).

Within the leading edge of the species' range, we did not find evidence of local selection based on range‐wide genotype‐environmental associations, but did identify genomic differences between the edge and core populations in the leading‐edge outlier SNPs. As these loci have distinct gene ontologies compared to the GEA outliers, the selection forces of a range expansion into novel environments by climatic anomalies may differ from those associated with long‐term climatic gradients. Leading‐edge populations harbor unique evolutionary potential, which might be beneficial in subsequent range expansions and thus of high conservation value (Gibson et al. 2009; Hoffmann et al. 2017). As marine heatwaves continue to increase in frequency and intensity (Oliver et al. 2018), these leading‐edge populations may be important stepping stones for future range expansions, further warranting protection from potential harvesting (Huang et al. 2020).

Our findings provide a necessary backdrop for eco‐evolutionary feedbacks occurring at the leading edge, which we are currently exploring in *L. gigantea* by assessing phenotypic differences in dispersal potential, growth rates, and age/size of sex change, and if these traits are plastic or genomic in nature. Ultimately, integrative and interdisciplinary approaches such as these are needed to understand the balance between vulnerability to climate‐ and human‐driven impacts and adaptive potential to evolve and persist under such threats. While we find no evidence of harvesting pressure depleting genetic diversity across populations, restricting harvesting within range‐edge populations might be a viable conservation strategy to sustain high abundances within these evolutionarily important units.

## Conflicts of Interest

The authors declare no conflicts of interest.

## Supporting information


**File S1:** A matrix of Spearman's correlation coefficients between all environmental predictor variables.

**Data S1:** Sampling information, code and outputs of HGAM analyses, outputs from outlier detection analyses, gene ontology analyses, and principal component analyses.

## Data Availability

Raw genomic data can be found on the NCBI sequence archive under SRA BioProject accession number: PRJNA1075458. The demographic data is publicly available from online requests via this webpage: https://marine.ucsc.edu/explore‐the‐data/contact/index.html. The code to analyze the data is available on GitHub: https://github.com/esnielsen/Lottia‐adaptation.
