# Supplementary material for: The Effects of Latitudinal Gradients, Climatic Anomalies, and Size‐Selective Harvesting on the Adaptive Potential of an Intertidal Gastropod
Source: Evol Appl. 2025 Sep 25;18(9):e70159. doi: 10.1111/eva.70159 (PMC12461133; doi:10.1111/eva.70159)
Supplement: Supplementary file 2 — Data S1: Sampling information, code and outputs of HGAM analyses, outputs from outlier detection analyses, gene ontology analyses, and principal component analyses. [file EVA-18-e70159-s001.docx]

Supplementary Material File 1 for “The effects of latitudinal gradients, climatic anomalies, and size-selective harvesting on the adaptive potential of an intertidal gastropod”

Supplementary Material File 1 Contents

- Code for Hierarchical Generalized Additive Models
- Table S1: Sampling information
- Table S2: Isolation-by-environment analysis outputs
- Table S3: Outputs from the redundancy analyses
- Table S4: The number of outlier SNPs detected across methodologies
- Table S5: The number of outlier SNPs selected by at least two detection methods
- Table S6: Gene ontology output from the topGO enrichment analysis on the range-wide, environmentally-associated outlier SNPs
- Table S7: Gene ontology output from the topGO enrichment analysis on the leading-edge outlier SNPs
- Table S8: Gene ontology output from the topGO enrichment analysis on the harvesting-associated outlier SNPs
- Figure S1: Principal components analysis of the linkage disequilibrium-pruned SNP panel, with each population subsampled to the lowest sample size of 19 individuals
- Figure S2: Map of the sites used to model demographic patterns
- Figure S3: Principal components analysis of genomic variation in Lottia gigantea within the range-wide environmentally-associated SNPs for the distributional core and leading edge sites
- Figure S4: Principal components analysis and redundancy analysis plots of genomic variation within the leading-edge outlier loci for the distributional core and leading-edge sites
- Figure S5: Principal components analysis of genomic variation within the harvesting-pressure outlier loci
- Figure S6: Boxplots of expected heterozygosity between pairs of protected sites not vulnerable to harvesting versus unprotected sites vulnerable to harvesting
- Figure S7: Partial effect plots from the hierarchical generalized additive models investigating abundance trends in Lottia gigantea for all size classes over year and latitude
- Figure S8: Partial effect plots from the hierarchical generalized additive models assessing counts of individuals, partitioned by size class.

Code for the hierarchical generalized additive models investigating the effect of protection level and size (Model 1) and year and latitude (Model 2) on abundance trends.

Model 1:

size.prot.mod <- bam(count ~ interaction(size_bin, protection) + s(year, bs = "cr",k = 5) + s(year, by = size_bin, bs = "cr", k = 5) + s(year, by = protection, bs = "cr", k = 5) + s(year, by = interaction(size_bin, protection), bs = "cr", k = 5) + offset(log(plots_sampled)), data=LG_counts, family=tw, method="REML")

Model 2:

lat.year.mod <- gam(count ~ size_bin + s(latitude) + s(year)+ s(latitude,by=size_bin)+

ti(year, latitude, k=c(20, 20), m=2) +

ti(year, latitude, by=size_bin, k=c(20, 20), m=1),

data=LG_counts, family=nb(link="log"), method="REML", select=TRUE)

Table S1 – Sampling information, including site name, coordinates, sampling date, number of individuals sampled, and whether they are vulnerable to harvesting or not. Tissue was collected under the following permit: California Fish and Wildlife Department permit # S-191200004-19122-001

| **Site name** | **Latitude** | **Longitude** | **Date sampled** | **Individuals sequenced** | **Harvesting exposure** | **Protection Status** |
| --- | --- | --- | --- | --- | --- | --- |
| Kruse Ranch | 38.59 | -123.35 | 01/30/21 | 30 | Not vulnerable | Protected |
| Fort Ross | 38.52 | -123.27 | 12/12/20 | 30 | Vulnerable | Harvesting allowed |
| Bodega Marine Reserve | 38.32 | -123.08 | 11/29/20 | 30 | Not vulnerable | Protected |
| Dillon Beach | 38.26 | -122.97 | 11/12/20 | 30 | Vulnerable | Harvesting allowed |
| Hopkins | 36.62 | -121.90 | 01/13/22 | 30 | Not vulnerable | Protected |
| China Rock | 36.61 | -121.96 | 01/12/22 | 30 | Vulnerable | Protected |
| Soberanes Point | 36.45 | -121.93 | 06/26/21 | 30 | Vulnerable | Harvesting allowed |
| Vandenberg | 34.75 | -120.63 | 06/30/21 | 30 | Not vulnerable | Protected |
| Government Point | 34.44 | -120.45 | 11/15/21 | 30 | Not vulnerable | Protected |
| Carpinteria | 34.39 | -119.51 | 11/16/21 | 30 | Vulnerable | Harvesting allowed |
| Willows Anchorage | 33.96 | -119.75 | 11/17/21 | 30 | Vulnerable | Harvesting allowed |
| Intake Pipes | 33.45 | -118.49 | 11/30/21 | 30 | Not vulnerable | Protected |
| Shark Harbor | 33.39 | -118.48 | 12/02/21 | 30 | Vulnerable | Harvesting allowed |
| Scripps | 32.87 | -117.25 | 11/05/21 | 30 | Vulnerable | Protected |
| Cabrillo | 32.67 | -117.24 | 11/01/21 | 30 | Not Vulnerable | Protected |
| Punta Baja | 29.95 | -115.81 | 01/04/22 | 19 | NA | NA |
| Santa Rosaliita | 28.65 | -114.25 | 02/24/21 | 29 | NA | NA |
| Bahía Tortugas | 27.69 | -114.89 | 02/26/21 | 29 | NA | NA |
| Bahía Asunción | 27.13 | -114.30 | 02/25/21 | 27 | NA | NA |

Table S2 - Outputs from the isolation-by-environment mantel tests, showing the r^2^, p-values, and q-values from tests of each environmental predictor variable.

| **Predictor variable** | **Mantel test r^2^ value** | **p-value** | **q-value** |
| --- | --- | --- | --- |
| Diffuse attenuation | -0.18 | 0.83 | 0.83 |
| pH | 0.696 | 0.001 | 0.004 |
| Sea surface temperature | 0.218 | 0.013 | 0.026 |

Table S3 - Outputs from the redundancy analyses (RDAs), run on the entire SNP dataset (LD-pruned SNPs) as well as each outlier SNP dataset. The following metrics are shown for each RDA: adjusted r^2^, the significance of each predictor variable, and the significance of each RDA axis.

| **RDA model** | **Adjusted** r^2^ | **Diffuse attenuation** | **pH** | **Sea surface temperature** | **Harvesting vulnerability** | **RDA1** | **RDA2** | **RDA3** | **RDA4** |
| --- | --- | --- | --- | --- | --- | --- | --- | --- | --- |
| All SNPs | 0.074 | 0.217 | 0.006 | 0.001 | NA | 0.001 | 0.430 | 0.539 | NA |
| Range-wide outlier SNPs | 0.658 | 0.001 | 0.001 | 0.001 | NA | 0.001 | 0.001 | 0.002 | NA |
| Leading-edge outlier SNPs | 0.596 | 0.009 | 0.030 | 0.001 | NA | 0.001 | 0.002 | 0.597 | NA |
| Harvesting outlier SNPs | 0.030 | 0.035 | 0.067 | 0.006 | 0.070 | 0.003 | 0.342 | 0.895 | 0.618 |

Table S4 - The number of outlier SNPs detected by the three genotype-environment association tests, both total and per environmental predictor variable.

| **Environmental variable** | **BayPass** | **LFMM** | **RDA** |
| --- | --- | --- | --- |
| Diffuse attenuation | 14 | 1,545 | 861 |
| pH | 318 | 3,394 | 83 |
| Sea-surface temperature | 72 | 1,724 | 288 |
| Total | 404 | 6,663 | 1,232 |

Table S5 - The number of outlier SNPs that were selected by at least two detection methods, with the number of overlapping SNPs shown per pairwise model comparison.

| **Comparison** | **Number overlapping SNPs** |
| --- | --- |
| BayPass - LFMM | 261 |
| BayPass - RDA | 3 |
| LFMM - RDA | 749 |
| Total SNPs in outlier dataset | 1,013 |

Table S6 - Gene ontology (GO) outputs from the topGO enrichment analysis on the range-wide, environmentally-associated outlier SNPs. The GO ID, function, number of annotated and significant genes, and their ontology are shown. (BP = Biological Process, MF = Molecular Function, CC = Cellular Component).

| **GO.ID** | **Function** | **Annotated (# genes)** | **Significant (# genes)** | **Ontology** |
| --- | --- | --- | --- | --- |
| GO:0007156 | homophilic cell adhesion via plasma membrane adhesion molecules | 81 | 9 | BP |
| GO:0071526 | semaphorin-plexin signaling pathway | 9 | 3 | BP |
| GO:0030111 | regulation of Wnt signaling pathway | 11 | 3 | BP |
| GO:0001525 | angiogenesis | 7 | 3 | BP |
| GO:0006928 | movement of cell or subcellular component | 170 | 13 | BP |
| GO:0006468 | protein phosphorylation | 350 | 18 | BP |
| GO:0035556 | intracellular signal transduction | 281 | 18 | BP |
| GO:0007264 | small GTPase mediated signal transduction | 101 | 8 | BP |
| GO:0006913 | nucleocytoplasmic transport | 37 | 4 | BP |
| GO:0007018 | microtubule-based movement | 142 | 10 | BP |
| GO:0007154 | cell communication | 1085 | 47 | BP |
| GO:0007165 | signal transduction | 1047 | 45 | BP |
| GO:0023052 | signaling | 1078 | 46 | BP |
| GO:0051716 | cellular response to stimulus | 1258 | 51 | BP |
| GO:0050896 | response to stimulus | 1436 | 55 | BP |
| GO:0016310 | phosphorylation | 406 | 19 | BP |
| GO:0007166 | cell surface receptor signaling pathway | 173 | 12 | BP |
| GO:0001568 | blood vessel development | 7 | 3 | BP |
| GO:0001944 | vasculature development | 7 | 3 | BP |
| GO:0007155 | cell adhesion | 137 | 11 | BP |
| GO:0007167 | enzyme linked receptor protein signaling pathway | 33 | 5 | BP |
| GO:0007178 | transmembrane receptor protein serine/threonine kinase signaling pathway | 19 | 3 | BP |
| GO:0009653 | anatomical structure morphogenesis | 39 | 4 | BP |
| GO:0009719 | response to endogenous stimulus | 21 | 3 | BP |
| GO:0009966 | regulation of signal transduction | 115 | 9 | BP |
| GO:0009968 | negative regulation of signal transduction | 22 | 3 | BP |
| GO:0010646 | regulation of cell communication | 135 | 9 | BP |
| GO:0010648 | negative regulation of cell communication | 22 | 3 | BP |
| GO:0015931 | nucleobase-containing compound transport | 43 | 4 | BP |
| GO:0022610 | biological adhesion | 138 | 11 | BP |
| GO:0023051 | regulation of signaling | 138 | 9 | BP |
| GO:0023057 | negative regulation of signaling | 22 | 3 | BP |
| GO:0031503 | protein-containing complex localization | 20 | 3 | BP |
| GO:0035239 | tube morphogenesis | 7 | 3 | BP |
| GO:0035295 | tube development | 7 | 3 | BP |
| GO:0048514 | blood vessel morphogenesis | 7 | 3 | BP |
| GO:0048583 | regulation of response to stimulus | 128 | 9 | BP |
| GO:0048585 | negative regulation of response to stimulus | 23 | 3 | BP |
| GO:0048646 | anatomical structure formation involved in morphogenesis | 7 | 3 | BP |
| GO:0048731 | system development | 62 | 5 | BP |
| GO:0051056 | regulation of small GTPase mediated signal transduction | 51 | 5 | BP |
| GO:0051169 | nuclear transport | 39 | 4 | BP |
| GO:0070848 | response to growth factor | 15 | 3 | BP |
| GO:0070887 | cellular response to chemical stimulus | 44 | 4 | BP |
| GO:0071310 | cellular response to organic substance | 30 | 4 | BP |
| GO:0071363 | cellular response to growth factor stimulus | 15 | 3 | BP |
| GO:0071495 | cellular response to endogenous stimulus | 21 | 3 | BP |
| GO:0072359 | circulatory system development | 10 | 3 | BP |
| GO:0098609 | cell-cell adhesion | 84 | 9 | BP |
| GO:0098742 | cell-cell adhesion via plasma-membrane adhesion molecules | 81 | 9 | BP |
| GO:1902531 | regulation of intracellular signal transduction | 76 | 6 | BP |
| GO:0032559 | adenyl ribonucleotide binding | 960 | 50 | MF |
| GO:0030695 | GTPase regulator activity | 48 | 7 | MF |
| GO:0030551 | cyclic nucleotide binding | 7 | 3 | MF |
| GO:0017154 | semaphorin receptor activity | 9 | 3 | MF |
| GO:0004386 | helicase activity | 83 | 8 | MF |
| GO:0004714 | transmembrane receptor protein tyrosine kinase activity | 15 | 3 | MF |
| GO:0005509 | calcium ion binding | 483 | 24 | MF |
| GO:0031418 | L-ascorbic acid binding | 18 | 3 | MF |
| GO:0008569 | ATP-dependent microtubule motor activity, minus-end-directed | 22 | 3 | MF |
| GO:0019787 | ubiquitin-like protein transferase activity | 122 | 8 | MF |
| GO:0005544 | calcium-dependent phospholipid binding | 26 | 3 | MF |
| GO:0003777 | microtubule motor activity | 112 | 8 | MF |
| GO:0016702 | oxidoreductase activity, acting on single donors with incorporation of molecular oxygen, incorporati... | 24 | 3 | MF |
| GO:0030554 | adenyl nucleotide binding | 961 | 50 | MF |
| GO:0004713 | protein tyrosine kinase activity | 50 | 5 | MF |
| GO:0005524 | ATP binding | 948 | 49 | MF |
| GO:0016887 | ATPase activity | 232 | 16 | MF |
| GO:0008483 | transaminase activity | 20 | 3 | MF |
| GO:0019842 | vitamin binding | 90 | 7 | MF |
| GO:0016462 | pyrophosphatase activity | 612 | 29 | MF |
| GO:0004672 | protein kinase activity | 357 | 18 | MF |
| GO:0016818 | hydrolase activity, acting on acid anhydrides, in phosphorus-containing anhydrides | 618 | 29 | MF |
| GO:0016817 | hydrolase activity, acting on acid anhydrides | 618 | 29 | MF |
| GO:0032553 | ribonucleotide binding | 1283 | 59 | MF |
| GO:0032555 | purine ribonucleotide binding | 1274 | 58 | MF |
| GO:0017111 | nucleoside-triphosphatase activity | 599 | 28 | MF |
| GO:0035639 | purine ribonucleoside triphosphate binding | 1262 | 57 | MF |
| GO:0097367 | carbohydrate derivative binding | 1415 | 63 | MF |
| GO:0043167 | ion binding | 3158 | 123 | MF |
| GO:0017076 | purine nucleotide binding | 1275 | 58 | MF |
| GO:0043168 | anion binding | 1534 | 70 | MF |
| GO:0140096 | catalytic activity, acting on a protein | 1288 | 53 | MF |
| GO:0016787 | hydrolase activity | 1992 | 78 | MF |
| GO:0036094 | small molecule binding | 1770 | 72 | MF |
| GO:1901265 | nucleoside phosphate binding | 1648 | 64 | MF |
| GO:0000166 | nucleotide binding | 1648 | 64 | MF |
| GO:0003824 | catalytic activity | 4708 | 164 | MF |
| GO:0003724 | RNA helicase activity | 30 | 4 | MF |
| GO:0003774 | motor activity | 128 | 8 | MF |
| GO:0005096 | GTPase activator activity | 44 | 6 | MF |
| GO:0008047 | enzyme activator activity | 68 | 6 | MF |
| GO:0008081 | phosphoric diester hydrolase activity | 38 | 4 | MF |
| GO:0016701 | oxidoreductase activity, acting on single donors with incorporation of molecular oxygen | 25 | 3 | MF |
| GO:0016769 | transferase activity, transferring nitrogenous groups | 20 | 3 | MF |
| GO:0019199 | transmembrane receptor protein kinase activity | 21 | 3 | MF |
| GO:0031406 | carboxylic acid binding | 25 | 3 | MF |
| GO:0043177 | organic acid binding | 25 | 3 | MF |
| GO:0060589 | nucleoside-triphosphatase regulator activity | 55 | 7 | MF |
| GO:1990939 | ATP-dependent microtubule motor activity | 22 | 3 | MF |
| GO:0005875 | microtubule associated complex | 57 | 5 | CC |

Table S7 - Gene ontology (GO) outputs from the topGO enrichment analysis on the leading-edge outlier SNPs. The GO ID, function, number of annotated and significant genes, and their ontology are shown (BP = Biological Process, MF = Molecular Function).

| **GO.ID** | **Term** | **Annotated** | **Significant** | **Ontology** |
| --- | --- | --- | --- | --- |
| GO:0034660 | ncRNA metabolic process | 173 | 3 | BP |
| GO:0071705 | nitrogen compound transport | 250 | 4 | BP |
| GO:0005506 | iron ion binding | 128 | 3 | MF |
| GO:0003723 | RNA binding | 416 | 5 | MF |
| GO:0003712 | transcription coregulator activity | 57 | 3 | MF |
| GO:0140098 | catalytic activity, acting on RNA | 212 | 3 | MF |

Table S8 - Gene ontology (GO) outputs from the topGO enrichment analysis on the harvesting-associated outlier SNPs. The GO ID, function, number of annotated and significant genes, and their ontology are shown. (BP = Biological Process).

| **GO.ID** | **Term** | **Annotated** | **Significant** | **Ontology** |
| --- | --- | --- | --- | --- |
| GO:0035556 | intracellular signal transduction | 281 | 3 | BP |
| GO:0007165 | signal transduction | 1047 | 7 | BP |
| GO:0023052 | signaling | 1078 | 7 | BP |
| GO:0007154 | cell communication | 1085 | 7 | BP |
| GO:0050896 | response to stimulus | 1436 | 8 | BP |
| GO:0051716 | cellular response to stimulus | 1258 | 7 | BP |


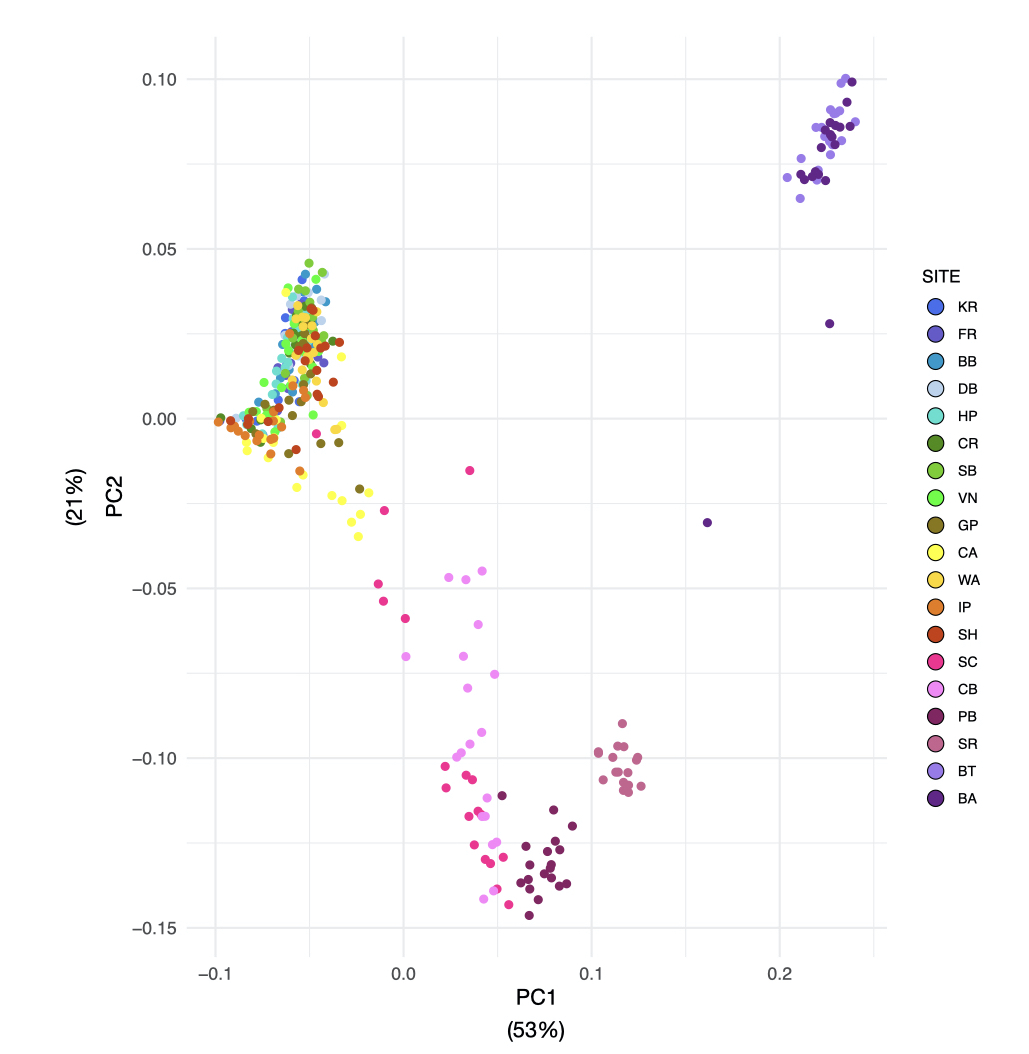


Figure S1 - Principal components analysis of the linkage disequilibrium-pruned SNP panel, with each population subsampled to the lowest sample size of 19 individuals. The PCA shows the same pattern as the non-subsampled dataset.
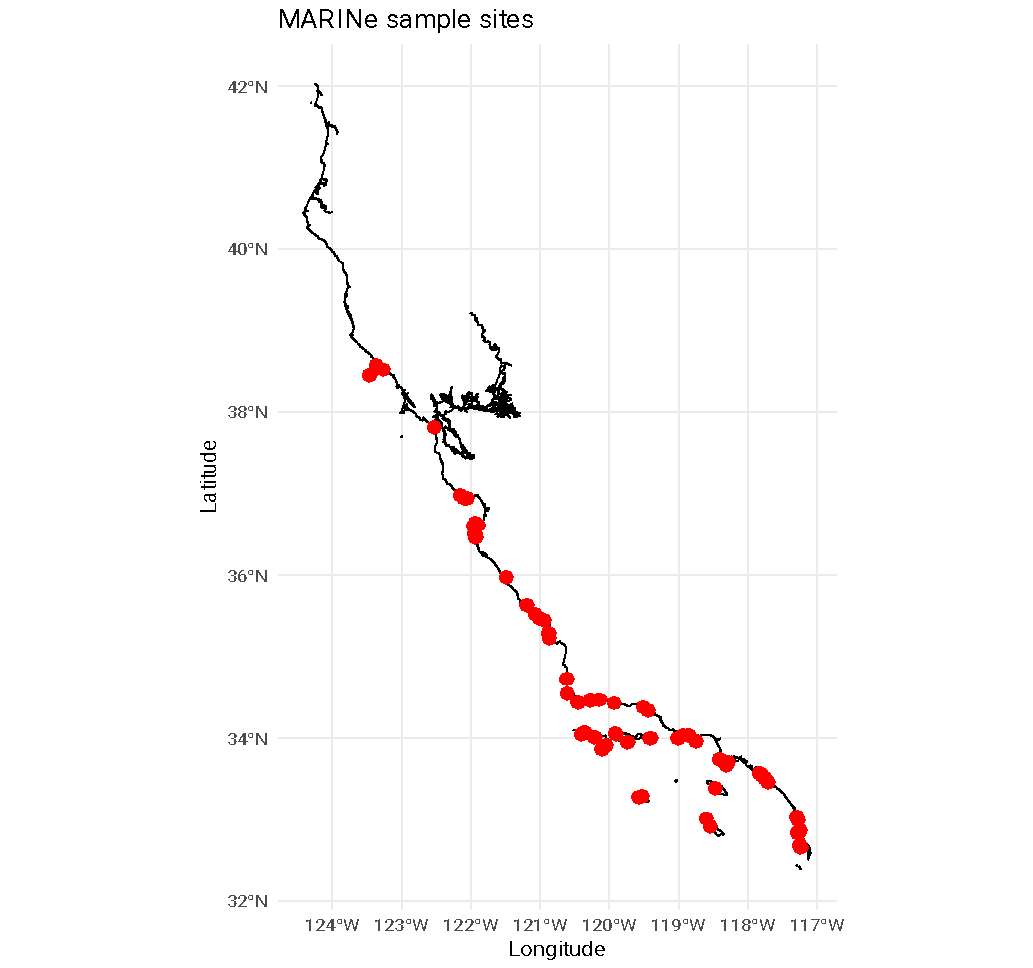


Figure S2 – Map of the 65 sites (indicated by red dots) along the California coast, sampled by the Multi-Agency Rocky Intertidal Network (MARINe) which included size counts for *Lottia gigantea* ([marine.ucsc.edu](http://eeb.ucsc.edu/) 2022) used to model demographic patterns.


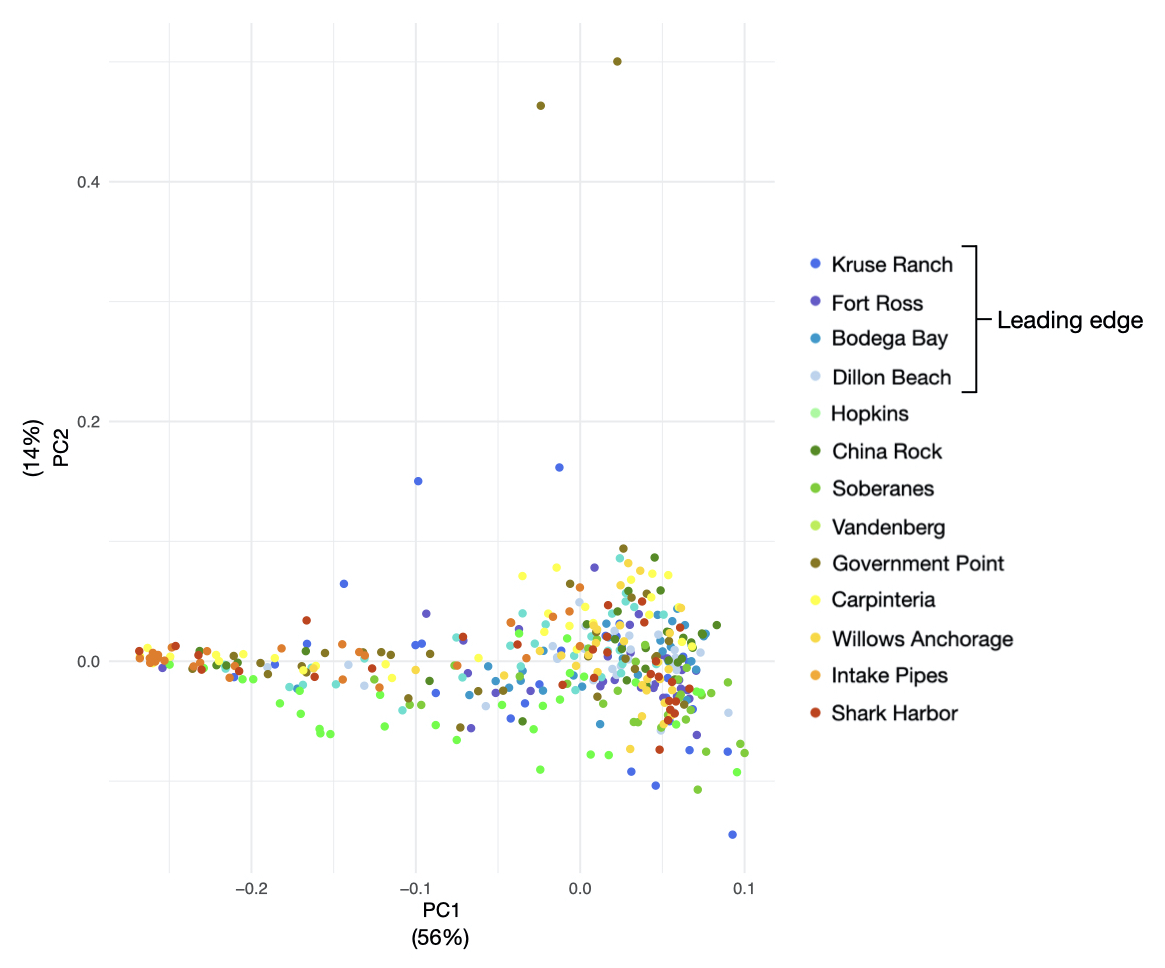


Figure S3 - Principal components analysis depicting genomic variation in *Lottia gigantea* within the range-wide environmentally-associated SNPs for the distributional core and leading edge sites.


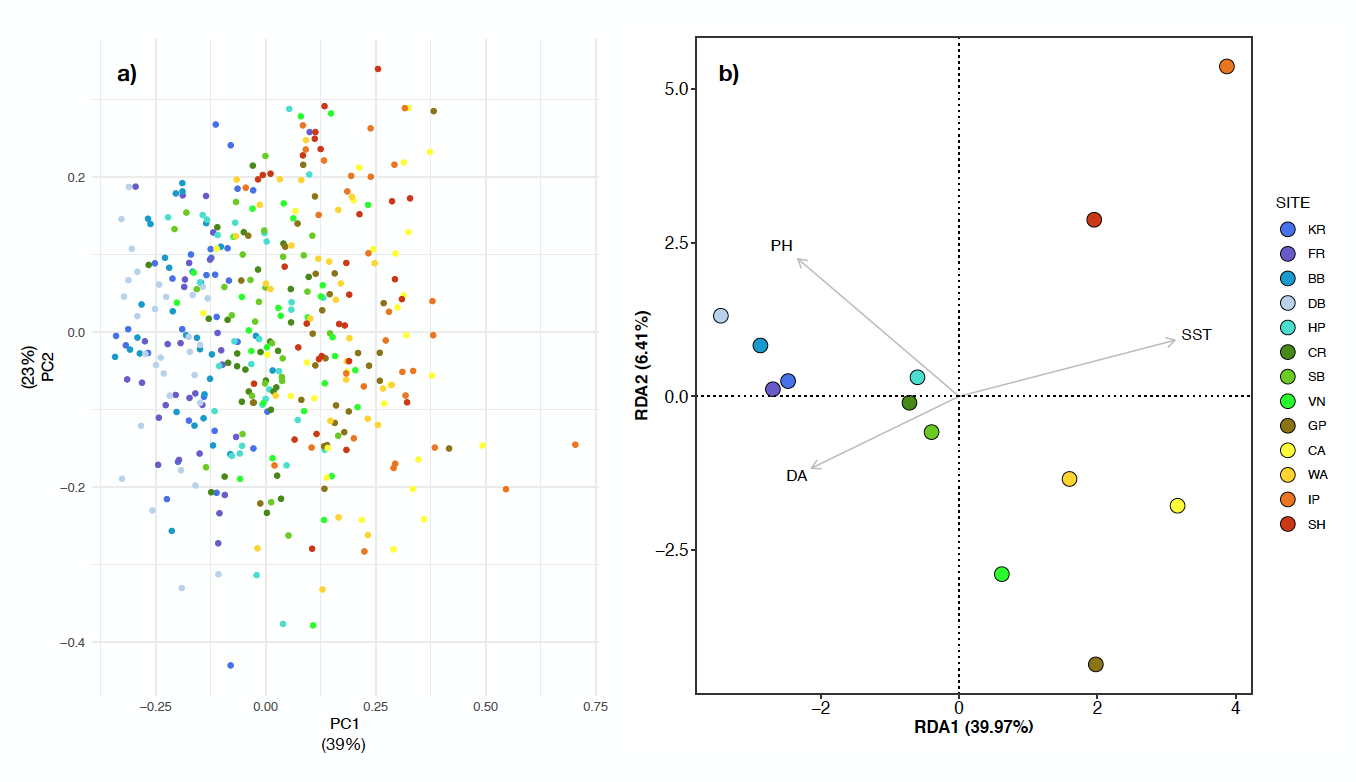


Figure S4 - Principal components analysis (a) and redundancy analysis (RDA) (b) plots depicting genomic variation within the leading-edge outlier loci for the distributional core and leading-edge sites. Within the RDA the sites are clustered based on their association with three environmental variables: diffuse attenuation (DA), pH, and sea-surface temperature (SST). Site abbreviations and locations are listed in Figure 2.


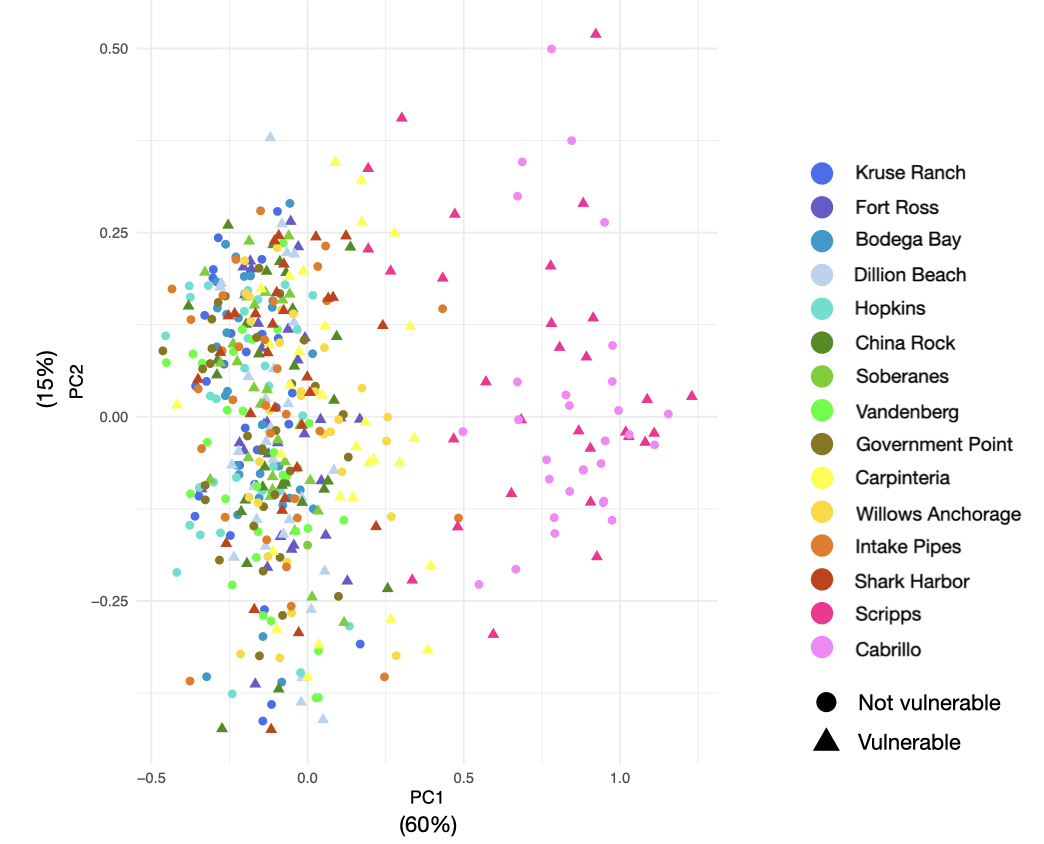


Figure S5 - Principal components analysis depicting genomic variation within the harvesting-pressure outlier loci. Sites not vulnerable to harvesting are indicated by circles and sites vulnerable to harvesting are indicated by triangles.


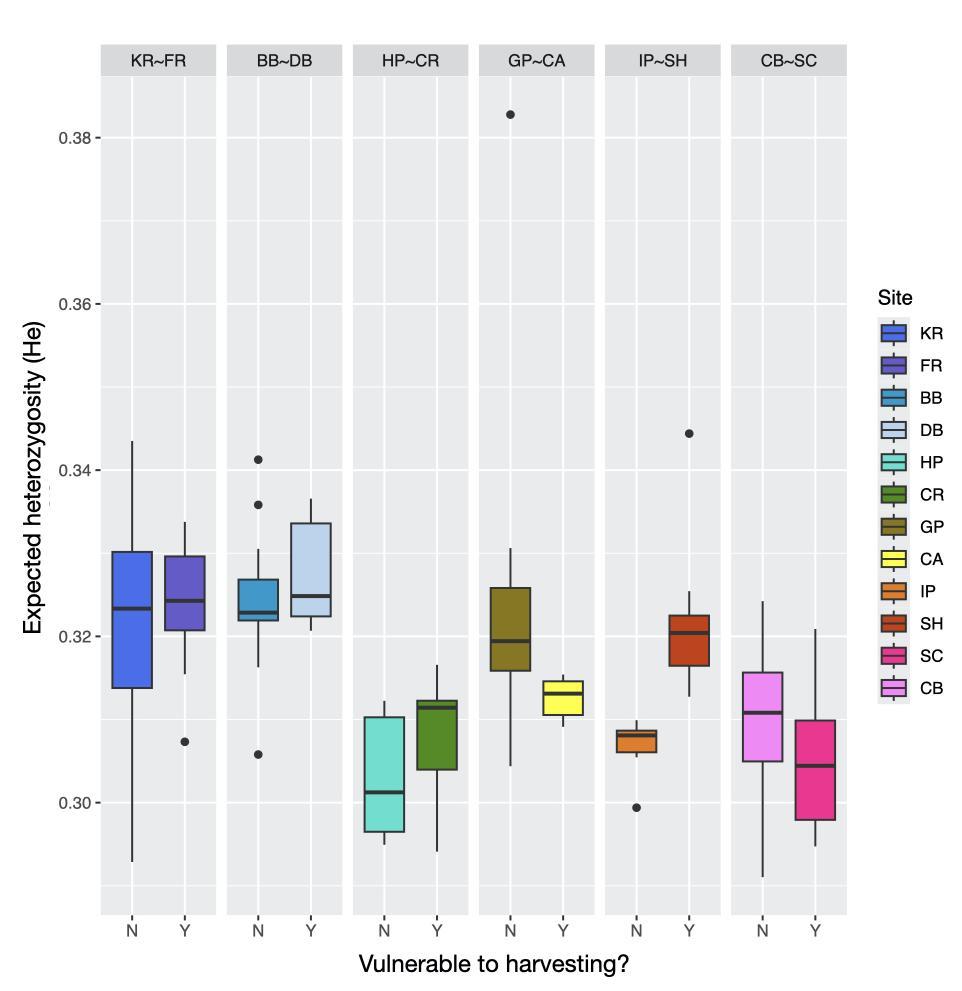


Figure S6 - Boxplots of expected heterozygosity (*H_e_*) between pairs of protected sites not vulnerable to harvesting (N) versus unprotected sites vulnerable to harvesting (Y). Data are for California sites from solely the large individuals (>41 mm). Site abbreviations and locations are listed in Figure 2.


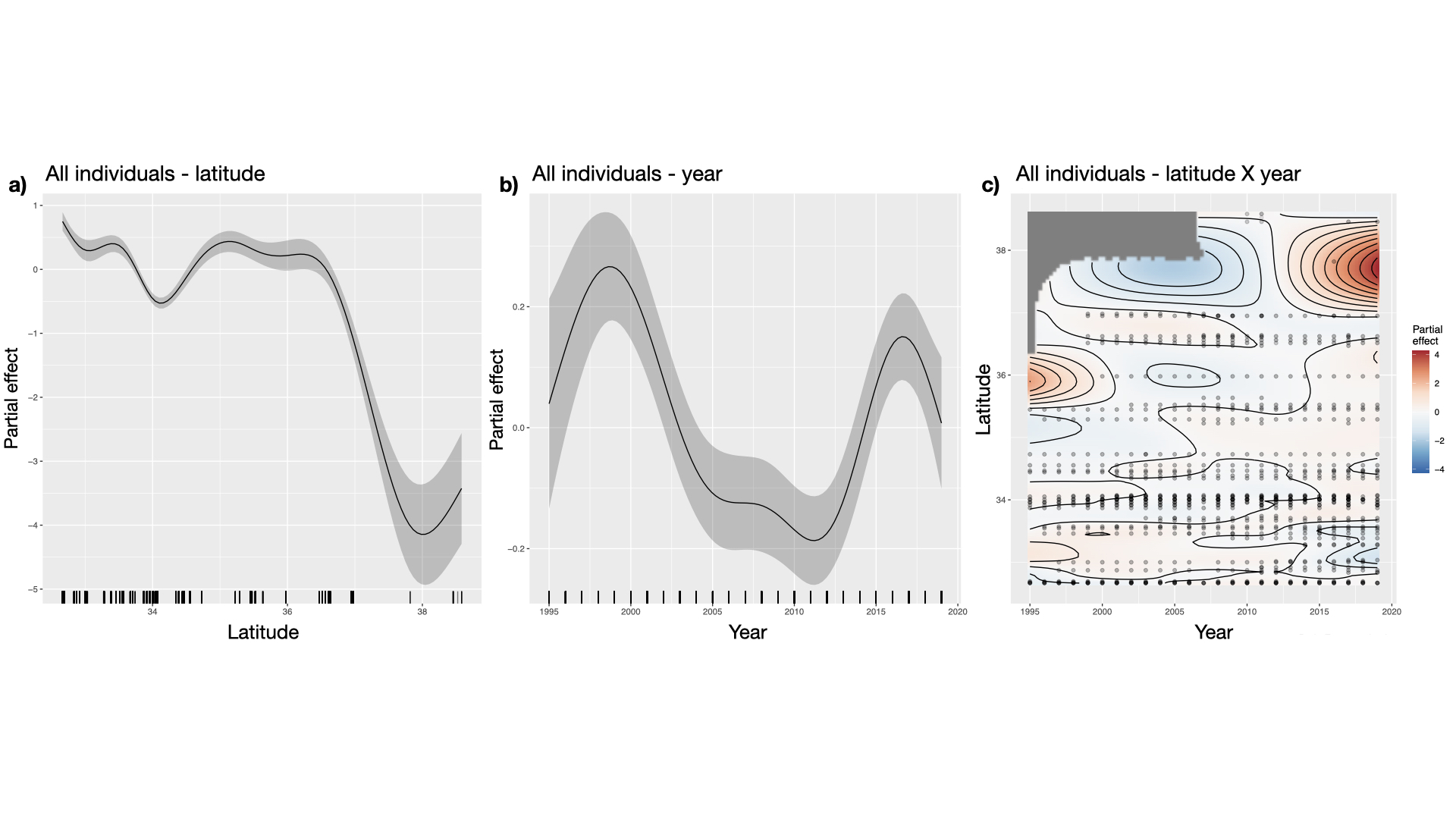


Figure S7 - Partial effect plots from the hierarchical generalized additive models investigating abundance trends in *Lottia gigantea* for all size classes over year and latitude. The plots show abundance trends over latitude (a), time (b), and across time and latitude combined (c). The tick marks on the x-axis in panel (a) indicate the latitudes of the sample sites included in the model. Abundance trends over time and latitude (c) show an increase in population size predominantly in higher latitudes during the years associated with the 1998 El Niño and 2014-16 marine heatwaves (areas in red represent higher abundance than otherwise expected, areas in blue represent lower abundance than expected, dark grey area indicates not enough data to make model predictions).


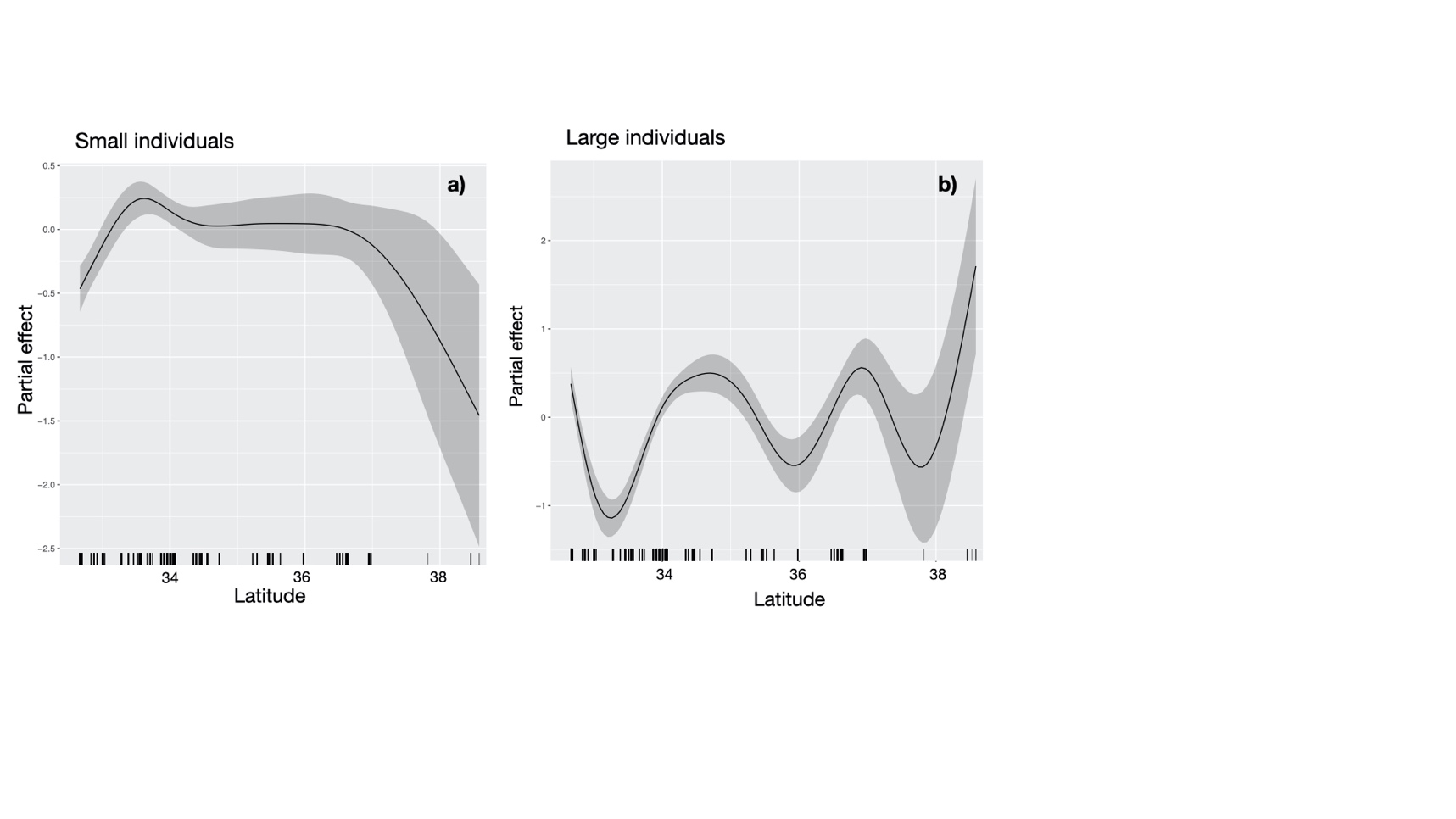


Figure S8 - Partial effect plots from the hierarchical generalized additive models assessing counts of individuals, partitioned by size class. The plots show abundance trends of (a) small individuals (<25mm) (a) and (b) large individuals (>41 mm) with x-axis ticks indicating Abundance of medium size individuals (26-40mm) is not shown and the model reported no significant effect of latitude on this size class.
